# A spatiotemporal computational model of focused ultrasound heat-induced nano-sized drug delivery system in solid tumors

**DOI:** 10.1080/10717544.2023.2219871

**Published:** 2023-06-14

**Authors:** Farshad Moradi Kashkooli, Mohammad Souri, Jahangir (Jahan) Tavakkoli, Michael C. Kolios

**Affiliations:** a Department of Physics, Toronto Metropolitan University, Toronto, ON, Canada; b Department of Nanobiotechnology, Pasture Institute of Iran, Tehran, Iran; c Institute for Biomedical Engineering, Science and Technology (iBEST), Keenan Research Centre for Biomedical Science, St. Michael’s Hospital, Toronto, ON, Canada

**Keywords:** Focused ultrasound, ultrasound-triggered nano-sized drug delivery system, solid tumor treatment, spatiotemporal modeling, bio-heat transfer, mass transport, stimuli-responsive drug-loaded nanocarriers, hyperthermia, thermo-sensitive liposome, drug release kinetics

## Abstract

Focused Ultrasound (FUS)-triggered nano-sized drug delivery, as a smart stimuli-responsive system for treating solid tumors, is computationally investigated to enhance localized delivery of drug and treatment efficacy. Integration of thermosensitive liposome (TSL), as a doxorubicin (DOX)-loaded nanocarrier, and FUS, provides a promising drug delivery system. A fully coupled partial differential system of equations, including the Helmholtz equation for FUS propagation, bio-heat transfer, interstitial fluid flow, drug transport in tissue and cellular spaces, and a pharmacodynamic model is first presented for this treatment approach. Equations are then solved by finite element methods to calculate intracellular drug concentration and treatment efficacy. The main objective of this study is to present a multi-physics and multi-scale model to simulate drug release, transport, and delivery to solid tumors, followed by an analysis of how FUS exposure time and drug release rate affect these processes. Our findings not only show the capability of model to replicate this therapeutic approach, but also confirm the benefits of this treatment with an improvement of drug aggregation in tumor and reduction of drug delivery in healthy tissue. For instance, the survival fraction of tumor cells after this treatment dropped to 62.4%, because of a large amount of delivered drugs to cancer cells. Next, a combination of three release rates (ultrafast, fast, and slow) and FUS exposure times (10, 30, and 60 min) was examined. Area under curve (AUC) results show that the combination of 30 min FUS exposure and rapid drug release leads to a practical and effective therapeutic response.

## 1. Introduction

The efficacy of conventional chemotherapy, as a mainstay of cancer treatment, is restricted by insufficient delivery of anti-cancer drugs to cancer cells and by toxicity to healthy tissue that limits the administered dosage. The former is primarily attributed to poor drug penetration from microvessels into the tumor interior arising from physiological obstacles of the tumor microenvironment (TME) and short circulation time (Jain, 1987). On the other hand, unfavorable deposition of drugs in healthy tissue can damage normal cells, causing a range of adverse effects. Different nano-sized drug delivery systems (DDSs) including liposomes, micelles, solid nanoparticles, and macromolecular drug carriers have been developed to overcome these restrictions. Stimuli-responsive nano-sized DDSs have recently illustrated great promise for cancer treatment to improve the drug bioavailability locally by providing controlled, timed, and destination-specific drug release (Mura et al., 2013; Karimi et al., 2016; Kashkooli et al., 2020; Mi, 2020; Kashkooli et al., 2021; Souri et al., 2022). The stimulus that facilitates drug release can be internal (such as pH, enzyme, hypoxia), external (such as magnetic, ultrasonic, light), or a combination of these (Gao et al., 2010; Karimi et al., 2016; Kashkooli et al., 2020; 2021; Van Durme et al., 2021). Most of these therapeutic agents are passive and aggregate in tumors due to the unique pathophysiology of the TME, including the large size of vessel wall pores that results in the enhanced permeability and retention (EPR) effect (Kashkooli et al., 2021; Moradi Kashkooli et al., 2021). The EPR effect is highly heterogeneous in different tumors. In addition, large drug carriers have a poor transvascular rate; hence, there is growing consensus that alternative methods are needed to improve targeting (Danhier, 2016; Ten Hagen et al., 2021). One proposed method is stimuli-responsive intravascular release (Ten Hagen et al., 2021), in which the encapsulated drug is released within the vessels and readily extravasated into the tumor. To achieve a high therapeutic response, the intravascular release paradigm should meet a basic principle: drug release at a high rate (Seynhaeve et al., 2020; Souri et al., 2022). External stimuli perform better for targeted rapid release within microvessels (Ten Hagen et al., 2021).

Among different external stimuli for activating drugs from drug-loaded NPs, ultrasound offers promising therapeutic results in both preclinical (Schultz et al., 2021) and clinical (Dimcevski et al., 2016) settings, by increasing localized uptake of therapeutic agents in solid tumors. Apart from common diagnostic uses, ultrasound has also been widely utilized in cancer treatments, including both destructive (i.e. thermal ablation) (Ter Haar, 2016) and nondestructive (i.e. mild hyperthermia) (Sirsi & Borden, 2014; Boissenot et al., 2016; Kashkooli et al., 2023) applications. Generally, FUS is capable of reaching deeper tissues while having minimal impact on the tissue between the surface of transducer and the focus area (Mihcin & Melzer, 2018). However, the heterogeneity of biological tissue can enhance complexity in ultrasound targeting of tumor areas (Zhang et al., 2022). Ultrasound-activated nano-sized DDSs have recently gained interest because they enable localized delivery of drugs to specific locations such as tumors (Couture et al., 2014; Ahmadi et al., 2020). Combining ultrasound with nano-sized DDSs can resolve some of the limitations of conventional nano-sized DDSs, including poor penetration into tumor tissue, the limited amount of drug released from NPs (Du et al., 2011), and loss of targeting ability. Ultrasound-nanocarrier interactions which lead to drug release are generally categorized as either thermal or mechanical interactions (Kashkooli et al., 2023). For any given application, a nanocarrier-drug compound can be engineered to react to a thermal excitation, mechanical stimulation, or a mix of the two to trigger drug release (Tharkar et al., 2019; Souri et al., 2022; Hornsby et al., 2023; Kashkooli et al., 2023). Therefore, ultrasound-induced DDSs can be designed to allow localized drug delivery into tumors while preserving normal tissues and organs at risk (Souri et al., 2022).

To enable ultrasound-activated release of drug, DDSs can be engineered with drug-loaded NPs that respond to ultrasonic waves (Hornsby et al., 2023). In the case of FUS-induced thermal fields, a mild temperature rise below the threshold of inducing any thermal tissue damage (<45 °C) can stimulate drug release from TSLs. This is the most well-known temprature-controlled drug-containing NPs reported in the literature (Kneidl et al., 2014), leading to enhanced drug and synergism with FUS in killing tumor cells (Papaioannou & Avgoustakis, 2022). Consequently, localized heating can be obtained through FUS, which is preferred in clinical thermal treatment due to its noninvasive nature, precision, and reliability (Hynynen, 2011). The safety and possibility of such a FUS-TSL hybrid delivery system were confirmed in recently published clinical human trials for treating liver tumors (Lyon et al., 2017; 2018; 2019). However, there is still an incomplete understanding of the mechanism of drug transport and drug uptake by tumor cells in response to FUS-induced temperature changes. In addition, heat absorption strongly depends on the thermophysical characteristics of the targeted tissue and the exposure parameters of the ultrasound beam (Izadifar et al., 2017). Hence, optimal treatment parameters should be chosen to increase the procedure’s efficacy, including providing the required temperature level, reducing the treatment duration, and inhibiting adverse effects on adjacent cells (Roohi et al., 2021).

Due to several stages included in the heat exchange and drug delivery process and the complex interactions of the ultrasound with tissue, the nanocarrier, and drugs, computational modeling plays a significant role in gaining in-depth insights into the delivery system and prediction of their therapeutic effectiveness. Early computational models in this field were developed to investigate interstitial fluid flow and macromolecule transport in solid tumors (Baxter & Jain, 1989; 1990; 1991). Subsequently, they expanded to several scales—including cellular and tissue—to examine the influences of various factors on drug transport (Wang et al., 1999; El-Kareh & Secomb, 2000; Goh et al., 2001; Eikenberry, 2009; Stylianopoulos et al., 2015; Zhan & Wang, 2018; Kashkooli et al., 2020; Löke et al., 2021; Moradi Kashkooli & Soltani, 2021; Soltani et al., 2021). Results of a one-dimensional drug delivery model showed that blood temperature had a significant influence on drug delivery outcome utilizing TSL (El-Kareh & Secomb, 2003). TSL performance was further analyzed compared with conventional chemotherapy and stealth liposomes under various circumstances (Gasselhuber et al., 2012), while a systemic parameter study was carried out to distinguish the most effective factors for determining maximum intracellular concentration by the TSL-high intensity focused ultrasound (HIFU) system (Liu & Xu, 2015). Gasselhuber et al. (Gasselhuber et al., 2012) combined the bio-heat transfer equation (BHTE) and a macroscopic drug transport model to predict the delivery of TSL and chemotherapeutic drugs under various heating regimens. To the best of our knowledge, no comprehensive mathematical modeling is presented in the literature that couples the physics of ultrasound and ultrasound-induced drug release kinetics with drug transport and delivery, especially for intravascular drug release. In addition, the temperature dependence of parameters associated with tumor tissue and therapeutic agents (TSL and chemotherapeutic agents) has not been rigorously investigated. More recently, a few groups attempted to address some of these issues for specific applications (Rezaeian et al., 2019; Zhan et al., 2019; Kim et al., 2021; Namakshenas & Mojra, 2023), but they do not present a comprehensive computational model that incorporates different physics, scales, and various details on the heterogeneous geometry of the tumor (e.g. microvasculature, necrotic core, hypoxic regions) in order to predict and optimize this DDS.

The main goal of the current study is to present a multi-scale and multi-physics computational model for FUS-triggered nano-sized DDS delivery in solid tumors. The present study proposes a model that predicts treatment responses by considering a wide range of biological interactions with drug agents in the intravascular release of DOX from TSL. Based on an extensive literature review and our best knowledge, this is one of the-first-of-its-kind studies to computationally examine FUS-triggered nano-sized DDS by integrating acoustic, heat transfer, intravascular drug release, interstitial fluid flow, mass transport, and bio-chemical activity of drug based on pharmacodynamics equations to examine treatment efficacy and AUC. For this purpose, we first examine the model’s validity in predicting the acoustic and thermal fields, which are the primary influencing factors on the TSL drug release. Subsequently, TSL/drug transport simulations are carried out in vascular, interstitial, and cellular spaces. Temperature-induced changes in tissue, TSL, and drug transport characteristics are also taken into account in the model. To quantify the effectiveness of treatment, pharmacodynamic parameters (i.e. the fraction of survival cancerous cells and the AUC) are calculated using intracellular and extracellular drug concentrations, respectively. In the following, two significant factors in FUS-induced nano-sized DDSs, drug release rate from TSLs and exposure time of FUS, are selected to examine their impacts on the AUC.

## 2. Materials and methods

### 2.1. Drug delivery mechanism

**Figure 1.**
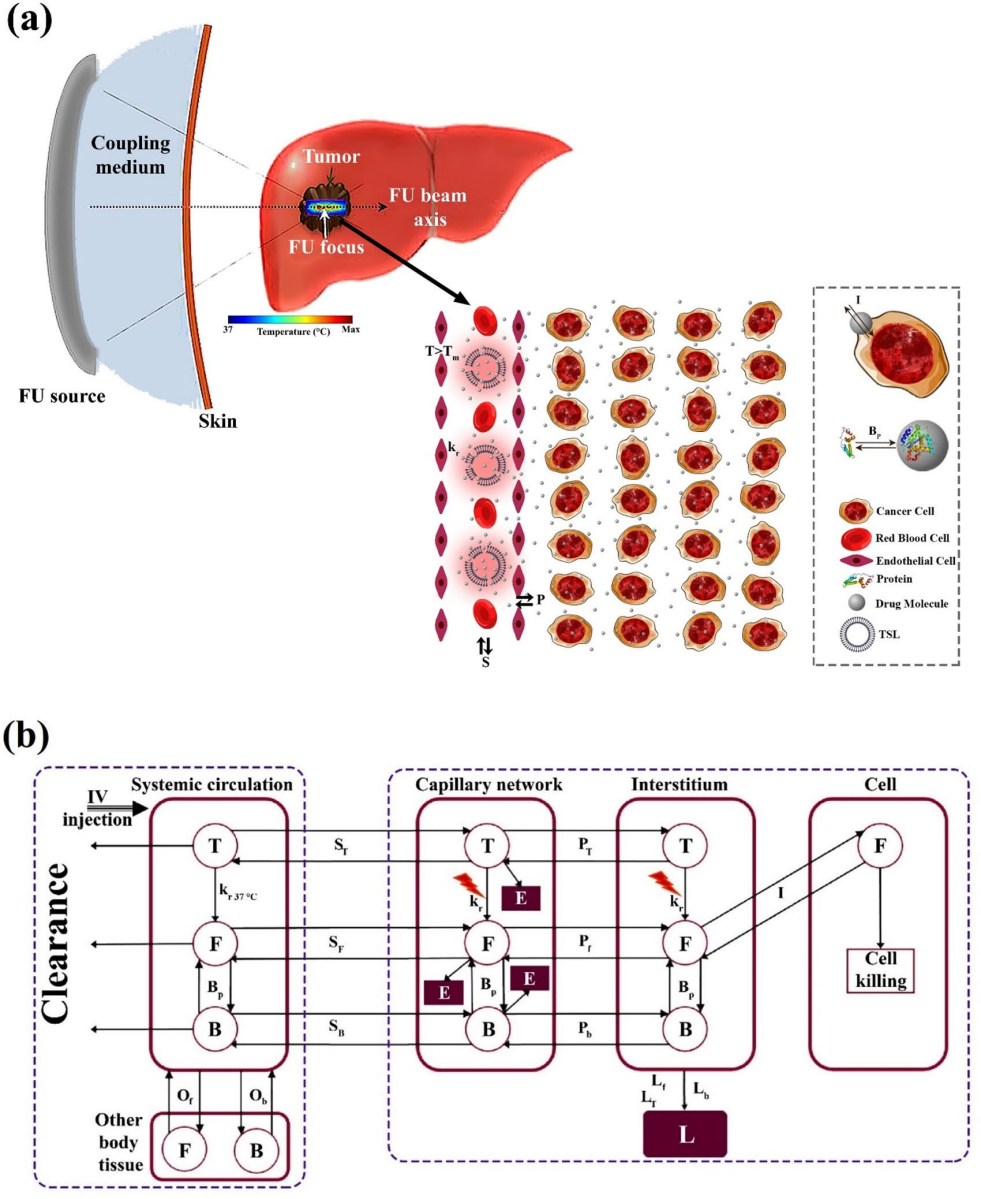
(a) Schematic of DDS and different interactions of TSL/drug agents. T, T_m_, k_r_, S, P, P_b_, and P_u_ Stand for temperature, melting temperature of TSLs, the release rate of the drug in response to the FUS-induced temperature rise, transport of drug agents between systemic circulation and microvessels, transport of drug agents between microvessels and interstitium, association/dissociation of drug molecules to protein, exchange of drug molecules between interstitium and cellular space, (b) multi-compartment model of the current study. T: TSL, F: free DOX, B: Protein-bound DOX, L: DOX removed by the lymphatic drainage system, E: Elimination in the microvascular network, S: Exchange between circulatory system and microvessels, P: Exchange between microvascular network and interstitium, I: Exchange between interstitium and intracellular, O: Exchange between systemic circulation and other tissue and organs, $k_{r\;37\mathrm{{}^{\circ}C}}$: Release rate of drug at 37 °C, $k_{r}$: Release rate of drug at temperature T, $B_{P}$: Association/dissociation rate of drug molecules to protein.

Schematics of the DDS and different interactions of TSL/drug agents, along with a multi-compartment model of the current study, are shown in Figure 1. Integration of TSL and FUS provides a DDS *via* drug release at the tumor site, where TSLs are administrated intravenously and reach the tumor site *via* the circulatory system. TSLs enter the tumor microvessels where thermally triggered drug release is initiated due to temperature increase in the sonicated region inside the tumor due to acoustic energy absorption. The released drug extravasates rapidly into the tumor tissue and is then taken up by tumor cells. Therapeutic drug molecules have the potential to bind to plasma proteins in the blood, preventing drug agents from targeting cancer cells (Greene et al., 1983). The assumption is that free drug molecules can enter the cellular space; on the other hand, free drug molecules can be pumped out of cells based on multidrug-resistant traits of cells (El-Kareh & Secomb, 2000; Chabner & Longo, 2011). The present mathematical model has been developed according to a multi-compartment model in which each bio-physical medium is considered a compartment, and therapeutic agents are exchanged between these compartments.

### 2.2. Mathematical modeling and governing equations

A detailed description of mathematical models, different parameters used in the models, and their references are listed in Table 1. First, the FUS-induced thermal field in the tissue and its microvessels is simulated. The acoustic wave propagation equation and BHTE are solved to simulate acoustic wave and thermal fields in the computational domain, respectively. Subsequently, a set of partial differential equations governing the TSL and DOX concentration distribution are solved, including the equations governing fluid flow in the interstitium and mass transport in systemic plasma and tissue. Darcy’s law, which describes the relationship between interstitial fluid velocity (IFV) and interstitial fluid pressure (IFP), is considered to define interstitial fluid flow in tissue as a porous medium. TSL transport and drug concentration in tissue can be determined through the convection-diffusion-reaction (CDR) equation. Upon entering the tissue interstitium, the free drug is taken up by cells through a passive mechanism driven by the drug concentration gradient. Intracellular drugs can be pumped out of cells, most probably through membrane transporter proteins involved in multidrug resistance (e.g. P-glycoprotein). Additionally, the free drugs binding to interstitial proteins (such as albumin) is also taken into account in the equations. Eventually, two pharmacodynamic models, which are dependent on internalized and extracellular concentrations, are used to calculate the cancer cells’ survival fraction and AUC, respectively.

**Table 1.** Different equations used in each step for modeling US-mediated nano-sized DDSs alongside their references.

	Sets of equations for	Equations	Parameters	Ref.
1	Acoustic waves propagation	Helmholtz wave equation for propagation of ultrasound beam in a medium	*I* : time-averaged acoustic intensity; *P* : acoustic pressure field; *c* : speed of sound; ρ : density; *f* : frequency; < > : time average over one acoustic cycle; *ω* : angular frequency; β : the absorption power; $\alpha_{0}$ : the absorption coefficient at $f_{0}\;=\;1\;\mathrm{MHz}$; The absorption coefficient changes linearly with the frequency for biological soft tissues (Solovchuk et al., 2013), therefore, β = 1.	(Roohi et al., 2021, Soltani et al., 2021, Rezaeian et al., 2019, Zhan et al., 2019,Zhan, 2014)
		$\frac{\kappa^{2}}{\rho }p+\nabla \cdot [\frac{1}{\rho }(\nabla p)]=0$ κ=ωc+iαABS		
		in which ω=2πf The absorption coefficient increases by the frequency enhancement and could be calculated by: $\alpha_{\mathrm{ABS}}=\alpha_{0}{\left(\frac{f}{{}_{0}}\right)}^{\beta }$		
		Ultrasonic power deposition per unit volume or external power deposition		
		$Q_{ex}=2\alpha I=\frac{2\alpha }{\omega^{2}\rho c}<{\left(\frac{\partial P}{\partial t}\right)}^{2}>$		
2	Temperature distribution	BHTE	*T* : temperature; *t* : tissue; *b* : blood; *ρ* : density; *c* : specific heat; *k* : thermal conductivity; $w_{bl}$ : capillary flow perfusion rate.	(Sedaghatkish et al., 2020; Kashkooli et al., 2021; Roohi et al., 2021; Souri et al., 2021)
		$\rho_{t}c_{t}\frac{\partial T_{t}}{\partial t}=k_{t}\nabla^{2}T_{t}-\underset{\text{The}\;\text{heat}\;\text{sink}\;\text{term}\;\text{due}\;\text{to}\;\text{blood}\;\text{perfusion}}{\underset{ï¸¸}{\rho_{bl}c_{bl}w_{bl}\left(T_{t}-T_{b}\right)}}+\underset{\text{The}\;\text{heat}\;\text{generated}\;\text{term}\;\text{by}\;\text{metabolism}\;\text{at}\;\text{the}\;\text{reference}\;\text{temperature}}{\underset{ï¸¸}{{\text{Q}}_{m}}}+\underset{\text{The}\;\text{external}\;\text{power}\;\text{deposition}\;\text{term}}{\underset{ï¸¸}{{\text{Q}}_{ex}}}$		
		PI controller to adjust an ideal range of temperature		
		$K_{\mathrm{HIFU}}=K_{p}(T_{\mathrm{set}}-T)+K_{i}\int_{0}^{t}(T_{\mathrm{set}}-T)dt$ $Q_{ex\_\mathrm{controller}}=K_{\mathrm{HIFU}}Q_{ex}$		
3	Interstitial fluid transport	Continuity equation	v : IFV; $p_{i}$: IFP; $F_{v}$ : source term that accounts for the gain of interstitial fluid from blood vessels; $F_{ly}$ : sink term that accounts for fluid absorption rate by the lymphatics; $K_{v}$ : hydraulic conductivity of the microvessel wall; $\frac{S}{V}$ : the surface area of blood vessels per unit tissue volume; $p_{v}$ : vascular pressures; p_ly_ : intra-lymph pressure; $\sigma_{T}$ : osmotic reflection coefficient; $\pi_{v}$ : osmotic pressure of the plasma; $\pi_{i}$ : osmotic pressure of the interstitial fluid.	(Moradi Kashkooli et al., 2021; Moradi Kashkooli & Soltani, 2021; Zhan, 2014; Souri et al., 2021)
		$\nabla \cdot v=F_{v}-F_{ly}$		
		$F_{v}=K_{v}\frac{S}{V}[p_{v}-p_{i}-\sigma_{T}(\pi_{v}-\pi_{i})]\;\&$ $F_{ly}=K_{ly}\frac{S_{ly}}{V}[p_{i}-p_{ly}]$		
		Conservation of momentum equation		
		$\frac{\partial (\rho v)}{\partial t}+\nabla .(\rho vv)=-\nabla p_{i}+\nabla .(\tau )-\frac{\mu }{\kappa }v$ which turns into Darcy’s equation as: $v=-\frac{\mu }{\kappa }\nabla p_{i}$		
4	Drug transport in tissue and cellular spaces	Liposome encapsulated drug concentration in circulation	*P*_l_ : liposome permeability of vasculature wall; *D*_l_ : liposome diffusion coefficient; σ_l_ : reflection coefficient for liposome; *CL*_lp_ : plasma clearance in tumor; *V*_T_ : total tumor volume; *V*_B_ : total blood volume in the body; $V_{BB}$: total blood volume in body; $H_{ct}$ : Hematocrit; $H_{\mathrm{ctt}\;}$: Hematocrit for tissue microvasculature; $V_{TV}$ : volume fraction of tissue vascular space; $\;v_{Tp}$ : volume fraction of microvessel plasma space; *P* : permeability of vasculature wall in tumor; *D* : diffusion coefficient in the interstitial fluid of tumor; σ_d_ : osmotic reflection coefficient; *k*_a_ : drug-protein binding rate; *k*_d_ : drug-protein dissociation rate; *φ* : tumor fraction extracellular space *V*_max_ : transmembrane transport rate; *k*_e_ : Michaelis constant for transmembrane transport; *k*_i_ : Michaelis constant for transmembrane transport; *CL* : plasma clearance in tumor	(Zhan, 2014; Zhan & Wang, 2018; Souri et al., 2021)
		VSp∂CLSP∂t=−CLLipsCLSPVSp−kr37CLSPVSp−FPTCLSPVTp+FPTCLTPVTp in which: $V_{Sp}=V_{BB}(1-H_{ct})$ $V_{Tp}=\;V_{T}\times v_{Tp}$ $v_{Tp}=\;$ ( $V_{TV}(1-H_{\mathrm{ctt}})$) $F_{PT}=\omega_{l}(1-{}_{\mathrm{ctt}})/v_{Tp}$		
		Liposome encapsulated drug concentration in tissue blood plasma		
		$\begin{array}{l} V_{Tp}\frac{\partial C_{LTP}}{\partial t}=-CL_{Lipt}C_{LTP}V_{Tp}-kr_{P}C_{LTP}V_{Tp}\\ -(F_{v}\left(1-\sigma_{l}\right)C_{LTP}+P_{L}\frac{S}{V}(C_{LTP}-C_{l})\frac{Pe_{l}}{e^{Pe_{l}}-1})V_{Tp}-\\ F_{PT}C_{LTP}V_{Tp}+F_{PT}C_{LSP}V_{Tp} \end{array}$ ; ${Pe}_{l}=\frac{F_{v}(1-\sigma_{l})}{P_{L}\frac{S}{V}}$		
		Liposome encapsulated drug concentration in the interstitial fluid		
		∂Cl∂t+∇⋅(Clvi)=Dl∇2Cle−krPCl+(Fv(1−σl)CLTP+PLSV(CLTP−Cl)PelePel−1)		
		Free DOX concentration in circulation		
		VSp∂CFSP∂t=kr37CLSPVSp+(kdCBSP−kaCFSP)VSp+(kftCFSPVBT−kfPCFSPVSp)−CLFSCFSPVSp−FPTCFSPVTp+FPTCFTPVTp		
		Free DOX concentration in tissue blood plasma		
		$\begin{array}{l} V_{Tp}\frac{\partial C_{FTP}}{\partial t}=kr_{P}C_{LTP}V_{Tp}-(F_{v}\left(1-\sigma_{d}\right)C_{fp}+\\ P_{fe}\frac{S}{V}(C_{fp}-C_{fe})\frac{Pe_{f}}{e^{Pe_{f}}-1})V_{Tp}+(k_{d}C_{BTP}-\\ k_{a}C_{FTP})V_{Tp}-CL_{ftp}C_{FTP}V_{Tp}-F_{PT}C_{FTP}V_{Tp}+\\ F_{PT}C_{FSP}V_{Tp} \end{array}$ ; ${Pe}_{f}=\frac{F_{v}(1-\sigma_{d})}{P_{fe}\frac{S}{V}}$		
		Free DOX concentration in interstitial fluid		
		∂Cfe∂t+∇.(Cfev)=Dfe∇2Cfe+Fv(1−σf)Cfp+PfeSV(Cfp−Cfe)PefePef−1−FlyCfe+kdCbe−kaCfe+ Dc Vmax(CiCi+ki−CfeCfe+keφ)+krelCle		
		Protein-bound DOX concentration in circulation		
		VSp∂CBSP∂t=+(kbtCBSPVBT−kbPCBSPVSp)−(kdCBSP−kaCFSP)VSp−CLBSCBSPVSp−FPTCBSPVTp+FPTCBTPVTp		
		Protein-bound DOX concentration in blood plasma (*C_bp_*)		
		$\begin{array}{l} V_{Tp}\frac{\partial C_{BTP}}{\partial t}=-(F_{v}\left(1-\sigma_{d}\right)C_{bp}+P_{be}\frac{S}{V}(C_{bp}\\ -C_{be})\frac{Pe_{b}}{e^{Pe_{b}}-1})V_{Tp}-CL_{btp}C_{BTP}V_{Tp}-(k_{d}C_{BTP}\\ -k_{a}C_{FTP})V_{Tp}-F_{PT}C_{BT}V_{Tp}+F_{PT}C_{BSP}V_{Tp} \end{array}$ ; ${Pe}_{b}=\frac{F_{v}(1-\sigma_{d})}{P_{be}\frac{S}{V}}$		
		Protein-bound DOX concentration in interstitial fluid		
		∂Cbe∂t+∇.(Cbev)=Dbe∇2Cbe+Fv(1−σb)Cbp+PbeSV(Cbp−Cbe)PebePeb−1−kdCbe+kaCfe		
		Intracellular DOX concentration (*C_i_*)		
		$\frac{\partial C_{i}}{\partial t}=\;V_{\max}(\frac{C_{fe}}{C_{fe}+k_{e}\varphi }-\frac{C_{i}}{C_{i}+k_{i}})$		
5	Pharmacodynamics model	Cell density variations	*D*_c_ : cell density; *f_max_* : cell-kill rate constant; *EC*50 : drug concentration creating 50% of *f_max_*; *k*_p_ : cell proliferation rate; *k*_g_ : cell physiologic degradation rate.	(Eliaz et al., 2004)
		$\frac{dD_{c}}{dt}=-\frac{f_{\max}C_{i}}{{EC}_{50}+C_{i}}D_{c}+{k_{p}D}_{c}-k_{g}{D_{c}}^{2}$		
		Area under the curve (AUC)	C(r,t): concentration in extracellular matrix; T : exposure time	(Kashkooli et al., 2020)
		$\mathrm{AUC}=\int_{0}^{T}C(r,\;t)dt$		

* The definition of the rest of parameters along with their values are available in the tables of the supplementary file.

### 2.3. Parameter values

Since the tumor and healthy tissue growth are negligible during drug delivery, all the geometric and drug transport parameters utilized in the present study are considered time-independent. Values adopted for different parameters and physics are summarized in Tables S1–S4. Physiological parameters, parameters for DOX, and parameters for the TSLs are listed in Tables S1–S3, respectively. In addition, since the temperature increase induced by ultrasound could affect some of the physical characteristics utilized in the drug delivery model, the temperature dependence of perfusion rate, thermal conductivity, specific heat capacity, density, vascular permeability, viscosity, diffusion coefficient, transmembrane rate are also considered (Table 2). Subsequently, acoustic parameters of the domain are presented in Table S4.

**Table 2.** Temperature dependency of some properties of tissue and drug alongside their references.

Parameters	Equations	Parameters	Refs.
Perfusion rate	$\omega =\omega_{0}\cdot DS$	ω : perfusion rate; Ω(t) : damage degree of biological tissue; C(0) : the initial concentration of healthy biological cells; C(t) : concentration of healthy biological cells remaining post thermal excitation; R : the universal gas constant; A : a frequency factor for the kinetic expression, ΔE : the activation energy of the thermal damage process, T : the instantaneous absolute temperature of the cells during thermal stress; t : time	(Schutt & Haemmerich, 2008)
	Degree of vascular stasis		
	$DS=e^{\text{-}\Omega \left(t\right)}$		
	Arrhenius model for calculating the damage degree of biological tissue:		
	$\Omega \left(t\right)=ln\left(\frac{C(0)}{C(t)}\right)=\overset{t}{\underset{0}{\int }}Ae^{\frac{-\Delta E}{RT(t)}}dt$		
Thermal conductivity	$k=k_{0}-0.02094T+3.89971\times 10^{-4}T^{2}-5.47541\times 10^{-7}T^{3}-4.14455\times 10^{-8}T^{4}+2.97188\times 10^{-10}T^{5}$	$k_{0}$ : thermal conductivity at $T_{0}$; $T_{0}$ : 37˚C	(Tan et al., 2018; Zou et al., 2020)
Specific heat capacity	$C=C_{0}+53.55552T-3.96009T^{2}+0.10084T^{3}-0.00106T^{4}+4.01666\times 10^{-6}T^{5}$	$C_{0}$*:* Specific heat capacity at $T_{0}$;	(Tan et al., 2018; Zou et al., 2020)
Density	$\rho =\rho_{0}-2.97434T+0.0042T^{2}+0.00293T^{3}-6.14447\times 10^{-5}T^{4}+3.33019\times 10^{-7}T^{5}$	$\rho_{0}$: Density at $T_{0}$;	(Tan et al., 2018; Zou et al., 2020)
Vascular permeability	For free and protein-bound drug	$P_{0}$ : permeability at $T_{0}$; P : permeability at T	(Zhan, 2014; Souri et al., 2021)
	$\frac{P_{0}}{P}=10^{0.7(T_{0}-T)}$		
	For TSL		
	$\frac{P}{P_{0}}=-10.54+5.76e^{\left(\frac{T-T_{0}}{5.44}\right)}+5.78e^{\left(\frac{T-T_{0}}{5.46}\right)}$		
Viscosity	$\frac{\mu }{\mu_{0}}=\exp(-0.03(T-T_{0})+1.04\times 10^{-4}(T^{2}-T_{0}^{2}))$	μ : viscosity at temperature *T*; $\mu_{0}$ : viscosity at temperature *T_0_*	(Zhan, 2014; Souri et al., 2021)
Diffusion coefficient	For free and protein-bound drug	$D_{0}$ : diffusivity coefficient of TSL at $T_{0};$ D : diffusivity coefficient of TSL at T	(Zhan, 2014; Souri et al., 2021)
	(No effect)		
	For TSLs		
	$\frac{D_{0}}{D}=\frac{T_{0}\mu }{T\mu_{0}}$		
Transmembrane rate	$k_{tv}=0.24T-7.88$	$k_{tv}$ : transmembrane rate	(Zhan, 2014; Souri et al., 2021)

*The definition of the rest of parameters along with their values are available in the tables of the supplementary file.

**Table 3.** Release rates of drug from TSLs at different temperatures (some data have been interpolated).

T (°C)		37	38	39	40	41	42
Krel (1/s)	Ultra-fast Release rates (for ThermoDOX) (Needham & Dewhirst, 2001; Gasselhuber et al., 2012; Centelles et al., 2018; Souri et al., 2021)	0.0003	0.0047	0.142	0.221	0.3	0.3
	Fast Release rates (for TSLs) (Tagami et al., 2012; Rezaeian et al., 2019)	0.00417	0.00545	0.0149	0.0282	0.0425	0.05409
	Assumed slow release rates (for TSLs)	0.000417	0.000545	0.00149	0.00282	0.00425	0.005409

### 2.4. Model geometry and boundary conditions

**Figure 2.**
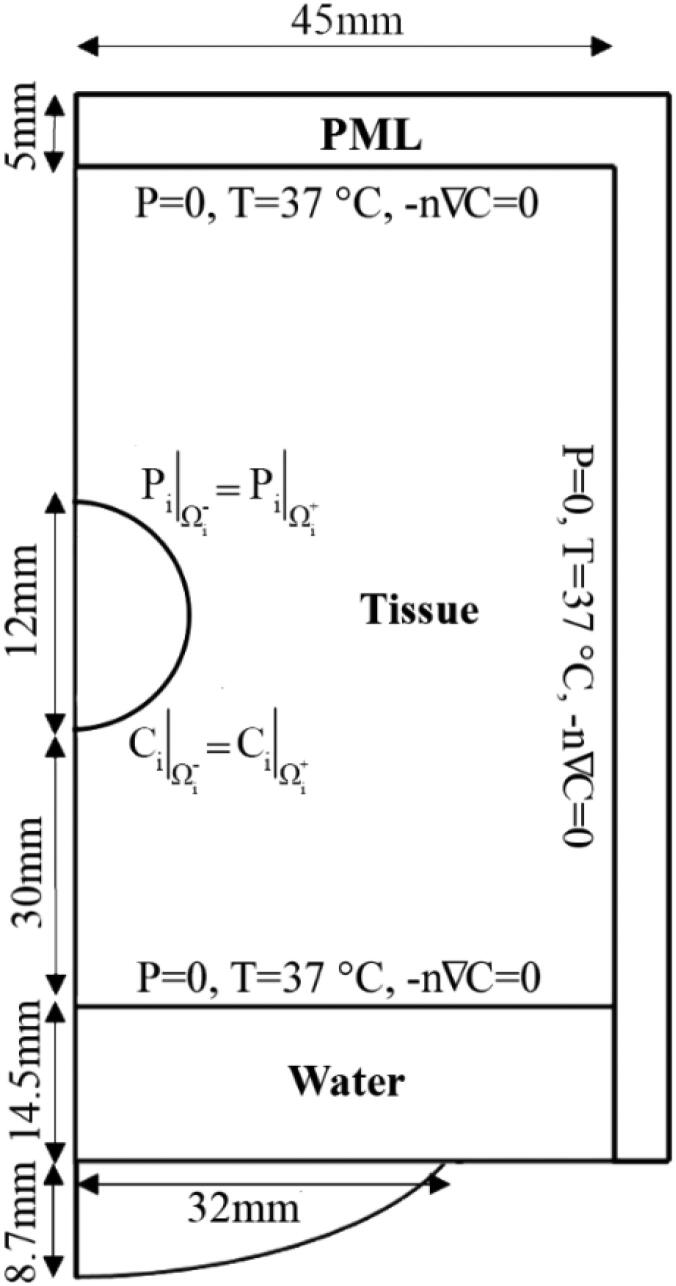
Schematic of the 2D axisymmetric computational domain (tumor, normal tissue, and transducer) and BCs.

A schematic of the computational domain used in this study, including the tumor, surrounding normal tissue, and transducer along with boundary conditions (BCs) are presented in Figure 2. A spherically focused 2.0 MHz single-element transducer, with 64.0 mm aperture diameter and 63.2 mm radius of curvature is selected in this simulation study. This transducer is located at the bottom side of the computational domain. As a baseline geometry, a spherical tumor with a radius of 12 mm is positioned in the center of a 2D block of normal tissue (72 × 45 mm^2^). The domain is surrounded by a 5-mm perfectly matched layer (PML) that uses artificial absorption to prevent waves from reflecting into the domain. In addition, BCs for acoustic, thermal and concentration simulations are shown in Figure 2. The COMSOL software defines the BCs between the tumor and healthy tissue while the user determines the other BCs.

### 2.5. Assumptions

Key assumptions considered in the simulation study are listed below:

- In this study, all simulations have been done using a two-dimensional axisymmetric geometry to reduce computational costs.
- The tumor is assumed to be well-vascularized with a uniform distribution of microvessels in the computational domain. Therefore, the tumors are not hypoxic. Despite this simplification, this is a widespread assumption in modeling drug delivery to solid tumors in the literature (Kashkooli et al., 2020; Soltani et al., 2021; Souri et al., 2021).
- Since ultrasound is not pulsed, we employ a frequency domain solution, which determines the steady-state acoustic field according to the operating circumstances and BCs. It is worth mentioning that non-linear effects and shear waves induced by ultrasound are ignored in the present study.
- There is always the possibility of sonoporation with or without ultrasound contrast agents. However, we have focused on the thermal effects caused by ultrasound in this study, and any mechanical effects (including sonoporation) have not been considered.
- Simulation time for ultrasound-mediated drug delivery is much shorter than the time scale for tumor growth; therefore, geometry and physiological variables are considered time-independent.
- In intravenous administration, it is considered that a perfect mixing within the systemic plasma happens, meaning that the entire body is exposed to the drug post-administration (Baxter & Jain, 1989; Gasselhuber et al., 2012; Souri et al., 2021). Therefore, the body is considered to be subjected to the administered drug concentration at time zero.
- DOX-loaded TSL is considered to mix perfectly and homogenously inside the plasma during administration.
- Only free DOX drug molecules can enter the cellular space and induce DNA damage and kill cancer cells. Efflux pumps wash out inactive drug molecules within the cellular space (Ughachukwu et al., 2012).

### 2.6. Numerical mesh size study

**Figure 3.**
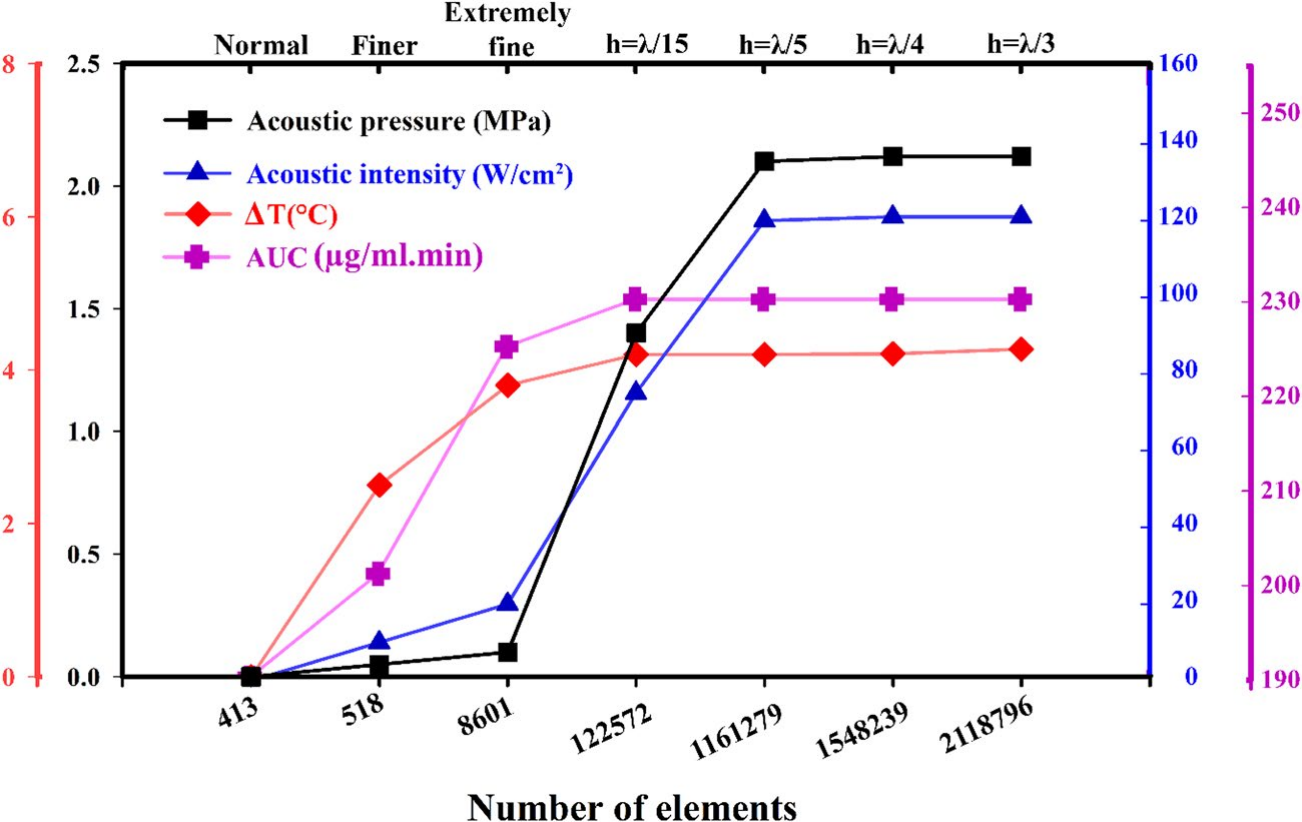
Grid independency test for acoustic pressure, acoustic intensity, temperature difference, and area under curve (AUC) parameters.

To correctly simulate the results, it is necessary to check the independence of the results from the mesh. The acoustic pressure and acoustic intensity are selected for the mesh analysis. Triangular elements discretize the entire domain. It is worth mentioning that the mesh independence test is also carried out during the mesh size selection. Seven meshes changing from normal to ultra-fine meshes are considered. Meshes with a smaller number of elements provide an inaccurate estimation of the acoustic field. After increasing the number of elements to 1,161,279, there is no significant change in the results, so this mesh is selected as the optimum mesh to evaluate the acoustic field for this study (see Figure 3). In this case, a maximum mesh element size is *h* = *λ*/5 (in which *λ*= wavelength and *h*= minimum size).

For heat transfer modeling in the computational domain, a mesh element size of *h* = *λ*/15 is chosen for the grids in the tumor area, the same morphology as the grid generation for acoustic modeling but in lower density, whereas in the surrounding healthy tissue, the mesh size gradually increases from *h* = *λ*/35 at tumor border to decrease the computational costs. The time-step is adjusted to 0.01 [s] due to the results of grid independence tests. For concentration (or AUC) analysis, triangular elements have also been used. Like temperature analysis, the *h* = *λ*/35 case with a total number of 122,572 cells is selected for solving drug transport equations because of its acceptable accuracy and capability to reduce computational costs (Figure 3).

### 2.7. Solution strategy and simulation cases

**Figure 4.**
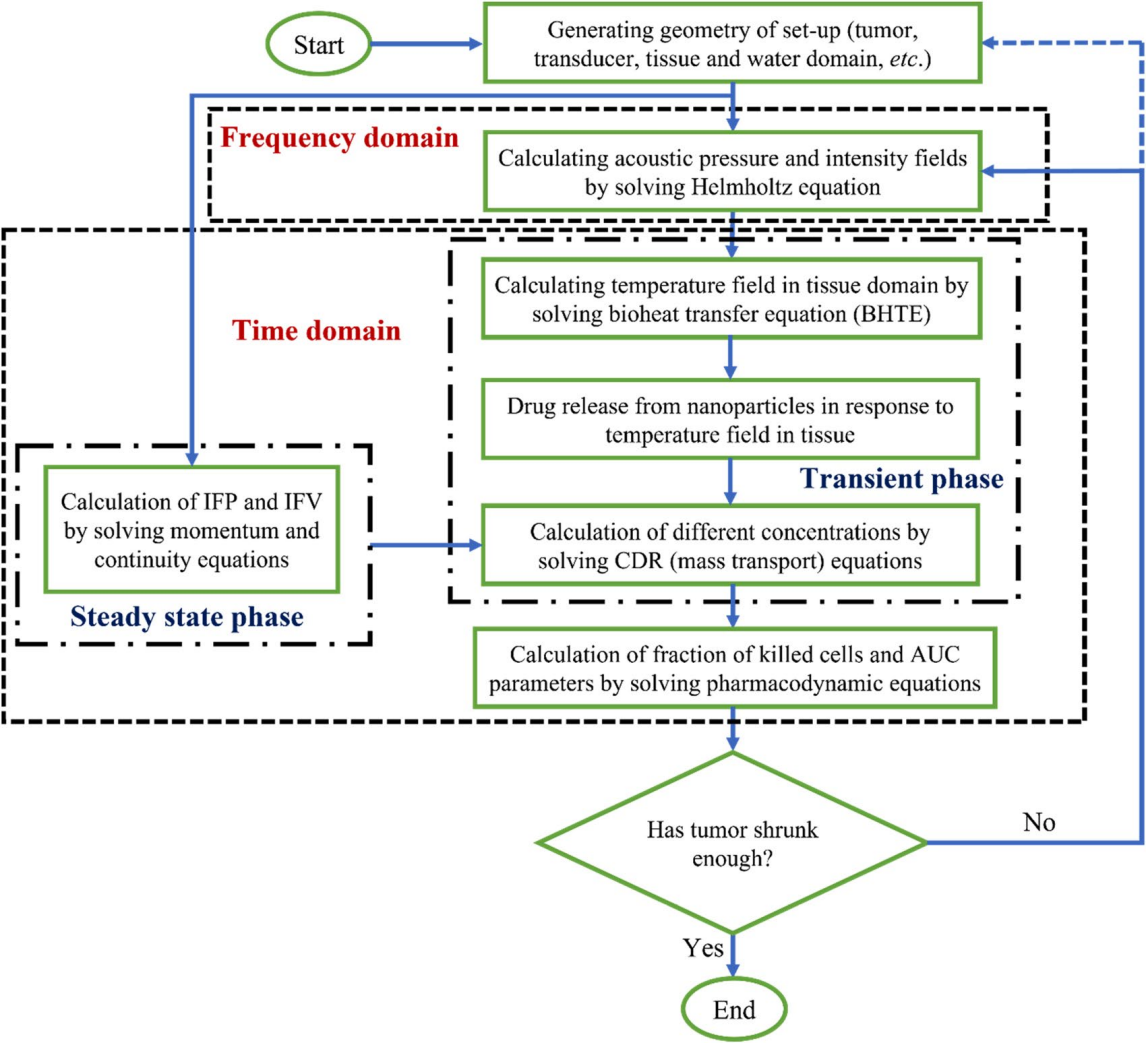
A step-by-step flowchart of the process for Assessing FUS-mediated nano-sized DDS in the present study.

The commercial finite element simulation tool COMSOL Multiphysics™ 6.0 (COMSOL Multiphysics Modeling Software, Stockholm, Sweden) is employed to model FUS-triggered nano-sized TSLs. A 2D-axisymmetric computational domain is simulated to attain a computation time in a reasonable range. The coupled acoustic and thermal fields calculate the temperature distribution by simultaneously solving the Helmholtz equation and BHTE. Frequency domain and time-dependent parameters were used. After determining acoustic and thermal fields, to obtain drug concentrations, there are two distinct solution phases in the current study: steady-state and transient. First, IFP and IFV are achieved *via* the momentum and continuity equations for interstitial fluid flow in the steady-state phase. Then, in a transient phase, distributions of different drug concentrations (*C_L_*, *C_F_*, *C_B_*) are obtained by solving mass transport equations, as listed in Table 1. The injected dose of TSL is 0.019 [kg/m^3^], which carries drug DOX molecules and is delivered through the intravascular release paradigm. Subsequently, the survival fraction of cancerous cells is calculated by solving a pharmacodynamic equation based on intracellular drug concentration. Figure 4 demonstrates a step-by-step flowchart of this process. An AMD Ryzen 9 5950 × 16-Core 3.40-GHz Processor with 64 GB RAM was used for the computational simulations.

### 2.8. Drug release rate from nanocarriers

Mathematical models of ultrasound-induced drug release are typically empirically derived from experimental data (Hornsby et al., 2022). Published TSL formulations in the literature (Tagami et al., 2012) still enable some DOX agents to be released gradually at 37 °C, although the release is much faster in mild hyperthermia range (at around 42 °C) (Kashkooli et al., 2020; Haemmerich et al., 2023). Release rate constants at different temperatures between 37 to 42 °C are given in Table 3 for ultra-fast, fast, and normal TSL drug release. ThermoDOX with ultra-fast release is selected as the baseline in this study, and the fast and slow release will be investigated in section 4.6.

ThermoDOX is designed to rapidly release its content when heated. The composition of the liposome, the method used to prepare it, and the temperature at which it is heated all affect the actual release rate (Tagami et al., 2011). The release rate also needs to be very low in the circulation so that the nanocarrier remains in circulation for a prolonged period and side effects are minimized. According to the literature, ThermoDOX has good stability at body temperature with a release rate of 3 × 1 0^−4^ (s^−1^) (Centelles et al., 2018). ThermoDOX will be effective when it releases its contents rapidly at high temperatures. ThermoDOX can release its drug payload explosively at a temperature of 43 °C with a release rate of 0.3 (s^−1^) (Gasselhuber et al., 2012), in less than a minute at temperatures within their melting range at 39–40 °C (Needham & Dewhirst, 2001).

## 3. Validation of the computational model

**Figure 5.**
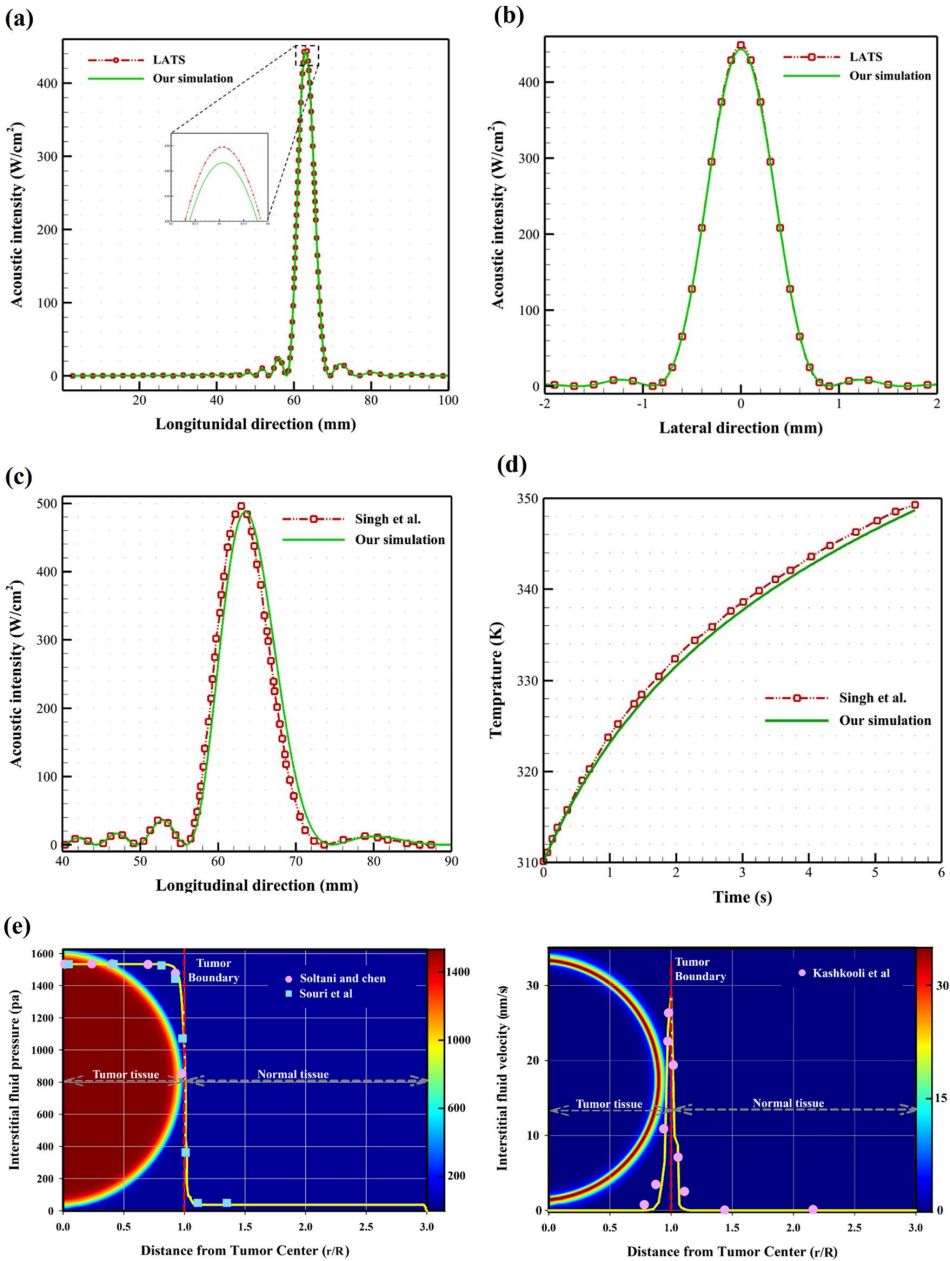
(a) and (b) A comparison of our results for acoustic fields with LATS in longitudinal and Lateral directions for water only. (c) and (d) Validation of acoustic and thermal fields of our simulation with Singh et al. (Singh et al., 2021), in which they applied FUS (15 W continuously for 5.6 s) on a domain containing water and muscle tissue. (e) Validation of the interstitial fluid flow (IFP and IFV) with those of literature (Soltani & Chen, 2011; Kashkooli et al., 2020; Souri et al., 2021). the yellow line and the contours in background (for spatial distribution of IFP and IFV) demonstrates our simulation results.

A validation of the developed computational model is presented in this section. As the model consists of several physics modules, each is compared separately with the previously published experimental or computational results to confirm the accuracy of numerical calculation and computational approach. As shown in Figure 5(a) and (b), we first compared our results for a geometry filled with water to LATS, a previously developed accurate software for linear acoustic and thermal simulations (Butt, 2011; Butt et al., 2011; Shaswary et al., 2021). Results show excellent agreement (with roughly a 1.33% discrepancy). Then, we selected a previously published study to assess the thermal field. Following the approach of Singh et al. (Singh et al., 2021), we first verified acoustic intensity in the geometry containing water and tissue (Figure 5(c)). Here, a concave transducer with a center frequency of 1 MHz, aperture diameter of 70 mm, and a 65 mm focal length was considered the FUS source with 15 W acoustic power and an exposure time of 5.6 s. In the following, the numerical methodology has been verified for the thermal field based on Pennes’ BHTE (Figure 5(d)). Results of temperature rise in the region of interest are in very good correspondence with Singh et al. (Singh et al., 2021), with a discrepancy of about 1.8%.

To validate the results of the present study for interstitial fluid flow (i.e. IFP and IFV), the studies conducted by Soltani and Chen (Soltani & Chen, 2011), Souri et al. (Souri et al., 2021), and Kashkooli et al. (Kashkooli et al., 2020) are examined for a tumor surrounded by healthy tissue, as demonstrated in Figure 5(e). IFP is a clinically important measure, particularly in tumor treatment using chemotherapeutic agents (Baxter & Jain, 1989). Additionally, IFP has a crucial role in the transport of therapeutic agents within the tumor extracellular matrix. The elevated IFP is one of the main barriers against effective delivery of drug to solid tumors by creating outward convection in the tumor borders versus inward diffusion (Moradi Kashkooli & Soltani, 2021). Overall, according to Figure 4(e), the highest IFP levels can be observed in the tumor due to the lack of a functional lymph drainage system in the tumor region as well as higher tumor permeability of vasculature (Jain & Baxter, 1988; Stylianopoulos et al., 2013). The results of the present study for the IFP prediction are in good agreement with those of Soltani and Chen (Soltani & Chen, 2011) and Souri et al. (Souri et al., 2021), as demonstrated in Figure 4(e). The average maximum IFP inside tumor agrees reasonably well with those in the computational analysis of Boucher et al. (Boucher et al., 1990), and also with the experimental outcomes of Huber et al. (Huber et al., 2005) and Boucher and Jain (Boucher & Jain, 1992). According to Darcy’s law, the IFV amount is proportional to the gradient of IFP. Because the gradient of IFP is distributed uniformly throughout the tumor, the IFV values is very low within the central zones of the tumor (Zhao et al., 2007; Pishko et al., 2011). The maximum IFV in our simulations occurs close to the tumor boundary, where the IFP reduction is steep, which matches the values in the previously published experimental and computational studies (Butler et al., 1975; Pishko et al., 2011). In addition, the results of this study for IFV have good correspondence with those of Kashkooli et al. (Kashkooli et al., 2020), as shown in Figure 5(e). The current study’s low IFV values are also in line with the recent *in vivo* investigation by Islam et al. (Islam et al., 2021). Therefore, the current model is considered reliable enough to predict the interstitial fluid flow characteristics in solid tumors.

## 4. Results and discussion

Results of the computational modeling of FUS heat-induced nano-sized DDSs are presented in this section. Acoustic and temperature profiles, drug transport in the tumor, and cellular drug uptake are shown in separate sections. Subsequently, two important factors in FUS-induced nano-sized DDSs, including the release rate of drug from TSLs and exposure duration of FUS, are also investigated in detail.

### 4.1. Acoustic and thermal field distribution

**Figure 6.**
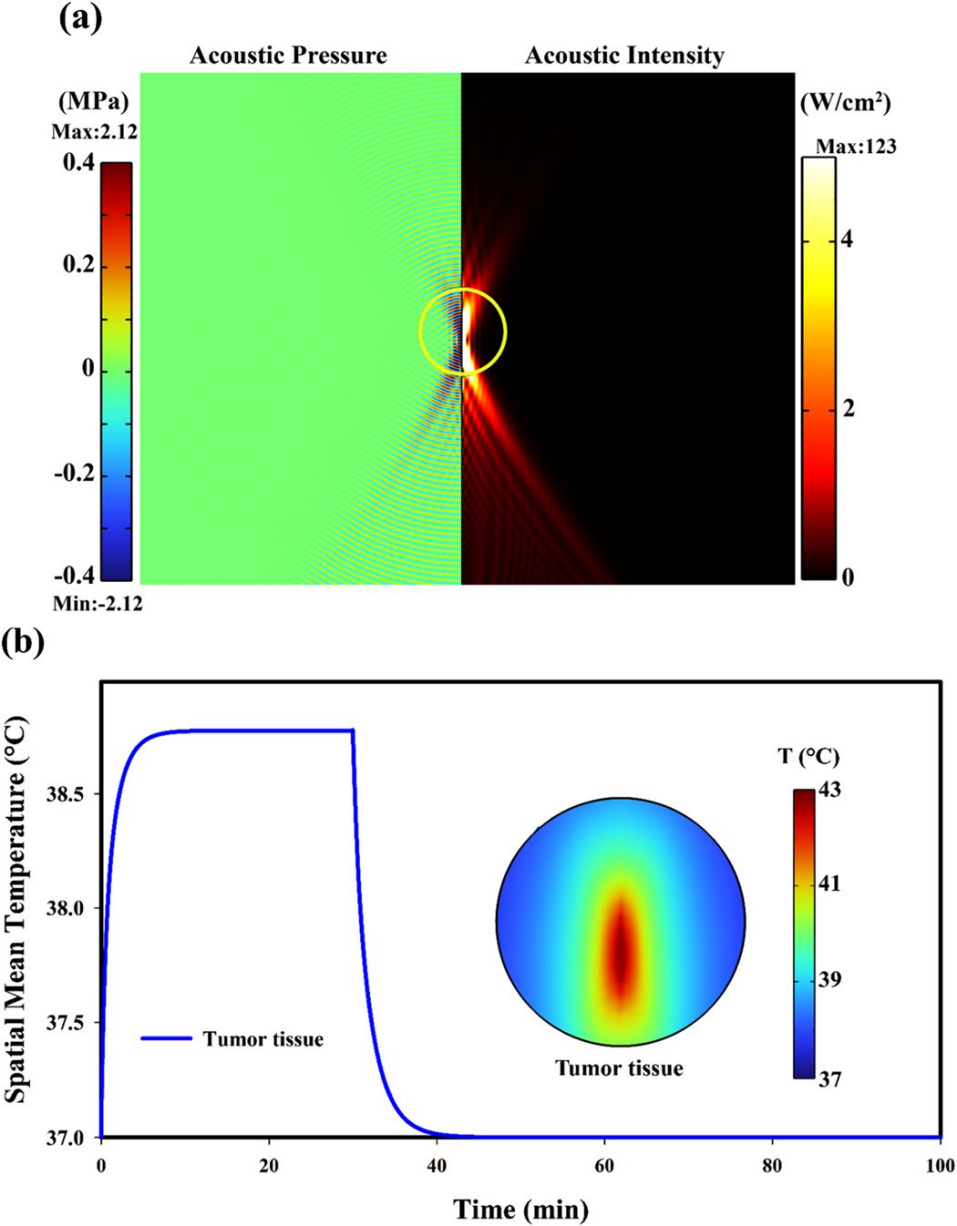
(a) the spatial distribution of acoustic intensity and pressure in the computational domain. It should be mentioned that since acoustic field is solved in a frequency domain, so the acoustic pressure and intensity profiles are not changing over time; (b) Spatiotemporal temperature distribution in tumor tissue (maximum value: 43 °C) considering temperature controller 15 min after appling FUS.

For a successful FUS-activated nano-sized DDS, ultrasonic energy should be delivered to a particular region of interest. In this work, the transducer’s focal point was placed at the tumor center. We presented acoustic intensity variations across the longitudinal direction in the entire water domain in section 3 in validation of the computational model. The distributions of ultrasound pressure and intensity throughout the simulated tissue domain for our main case study, which comprises water and tissue, are shown in Figure 6(a). The maximum amplitudes of the focal acoustic pressure and intensity are roughly 2.12 MPa and 123 W/cm^2^, respectively. However, in addition to the values of maximum pressure and intensity amplitudes, the ultrasound beam’s sufficient coverage of the target region of interest is another significant factor affecting the release of drugs in the targeted area. In our case study, ultrasonic energy covers almost the entire tumor region volume, where the diameter of the tumor is 12 mm.

The temperature profile in the focal area is elliptical, consistent with the focal spot shape from the ultrasound transducer. The temperature is calculated by solving the BHTE. To prevent thermal damage to the healthy tissue and blood vessels, by adjusting the input power, a PI controller has been utilized to achieve the desired temperature range in the ROI, i.e. to regulate the temperature into the hyperthermia range. Temperature variations induced by FUS heating over time in blood microvessels and tumor are shown in Figure 6(b). The maximum temperature of the tumor, which occurs at the focal point, quickly reaches 43 °C, and the mean temperature of the tumor reaches 40.4 °C, and it then remains at this level as long as the FUS transducer is on. The temperature drops gradually after the transducer is turned off. Since the focal area of FUS is directly positioned within the tumor and exposed to heating, tumor temperature increases faster than healthy tissue.

The spatial mean temperature of tissue at 20 min post-heating is demonstrated in Figure 6(b). The maximum temperature occurs in the focal area, where the tumor is directly exposed to the FUS. The increase in temperature in the surrounding area results from heat exchange *via* convection and conduction mechanisms from the focal area; thus, healthy tissue temperature is less than tumor temperature. The difference (2–3 °C) in spatial mean temperature between the tumor and healthy tissues increases drug release in the tumor area while minimizing the bioavailability of the drug in surrounding healthy tissues.

### 4.2. Drug transport in tumor

**Figure 7.**
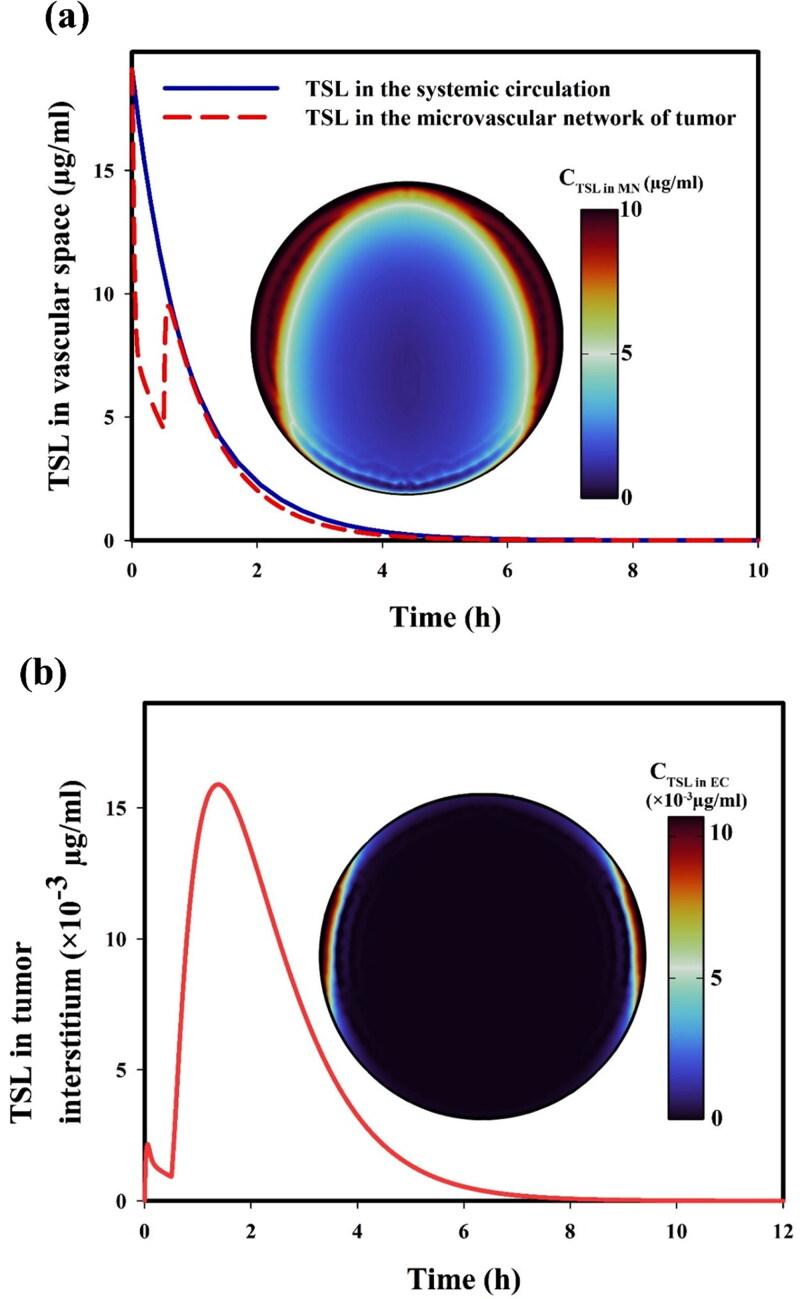
TSL concentration in (a) microvessels and (b) interstitium as a function of time. Note that the ultrasound exposure stops at t = 0.5 h.

Temporal profiles of TSLs in the systemic circulation, tumor microvessels, and tumor interstitium are shown in Figure 7. By bolus intravenous administration of drug-loaded TSL, the concentration of TSL at the initial moments in intravascular space is equal to the injection dose. As shown in Figure 7(a), the TSL concentration in systemic circulation gradually decreases, while TSLs experience different drug release kinetics in tumor microvessels, dependent on FUS heating time. The large vessel-wall pore size in the tumor site (50 nm (Stylianopoulos et al., 2015; Stylianopoulos & Jain, 2013)<d < 2 μm in diameter (McDonald & Baluk, 2002; Stylianopoulos & Jain, 2013; Stylianopoulos et al., 2015)) allows some TSLs to enter the TME through a passive mechanism (i.e. EPR effect), which increases TSL concentration in the tumor interstitium. This means that for large-size nanocarriers like TSLs, the concentration gradient term does not play a significant role in the transvascular exchange from microvessels into the interstitium due to the relatively low permeability. However, the convection term is much more significant since the IFP value in the periphery of the tumor is much lower than the intravascular pressure. Therefore, a small amount of TSLs enter the interstitium due to the plasma flow. For this reason, only a small number of TSLs had a chance to enter the central areas of the tumor, while the TSLs could enter the tumor periphery more readily. After a short time, due to the rapid drug release within tumor microvessels, almost no TSL could enter the tumor interstitium; however, after the microvessel cooling process at the end of the ultrasound exposure, some TSLs have the chance of entering the extracellular space. Subsequently, after reaching a maximum TSL concentration in the tumor interstitium, it gradually dropped to zero owing to the fast removal in the circulation system and increased drug release resulting from the temperature increase. In other words, the TSL concentration reaches its maximum and reduces progressively to zero due to drug release and reduced intravascular concentration (Figure 7(b)).

**Figure 8.**
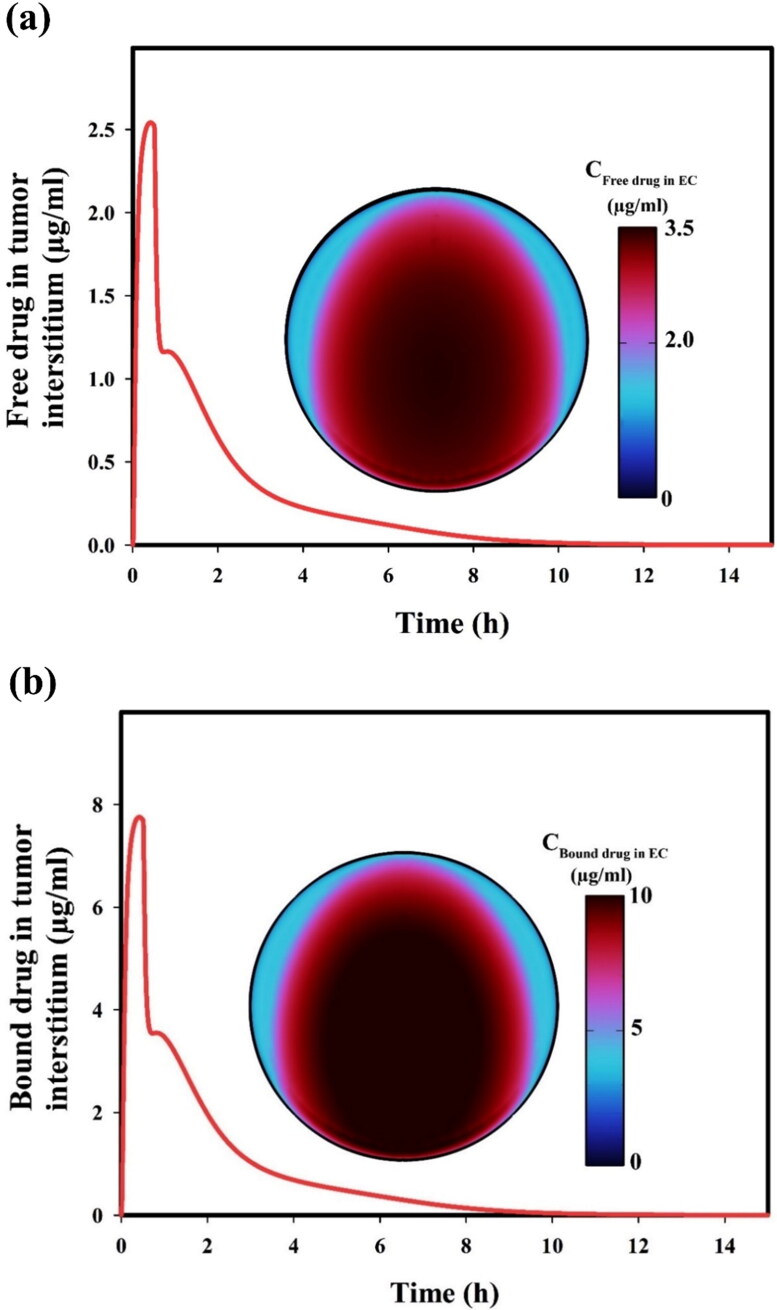
(a) Free and (b) protein-bound drug concentrations in the tumor interstitium as a function of time. Note that the ultrasound exposure stops at t = 0.5 h.

Free DOX is constantly released from TSLs by applying FUS heating, leading to a fast rise in drug concentrations, both in intravascular and extracellular spaces, followed by a gradual decrease in concentrations of the drug after reaching their maximum (Figure 8(a)). This is due to cancer cell drug uptake and the reduction in intravascular TSL concentration over time (Figure 7(a)). These results also reflect that temporal variation in the concentration of free drug (Figure 8(a)) and follows a similar trend as the concentration of protein-bound drug (Figure 8(b)), even though the concentration of the protein-bound drug is 2 to 3 times higher in magnitude than free drug concentration.

### 4.3. Cellular uptake of drug

**Figure 9.**
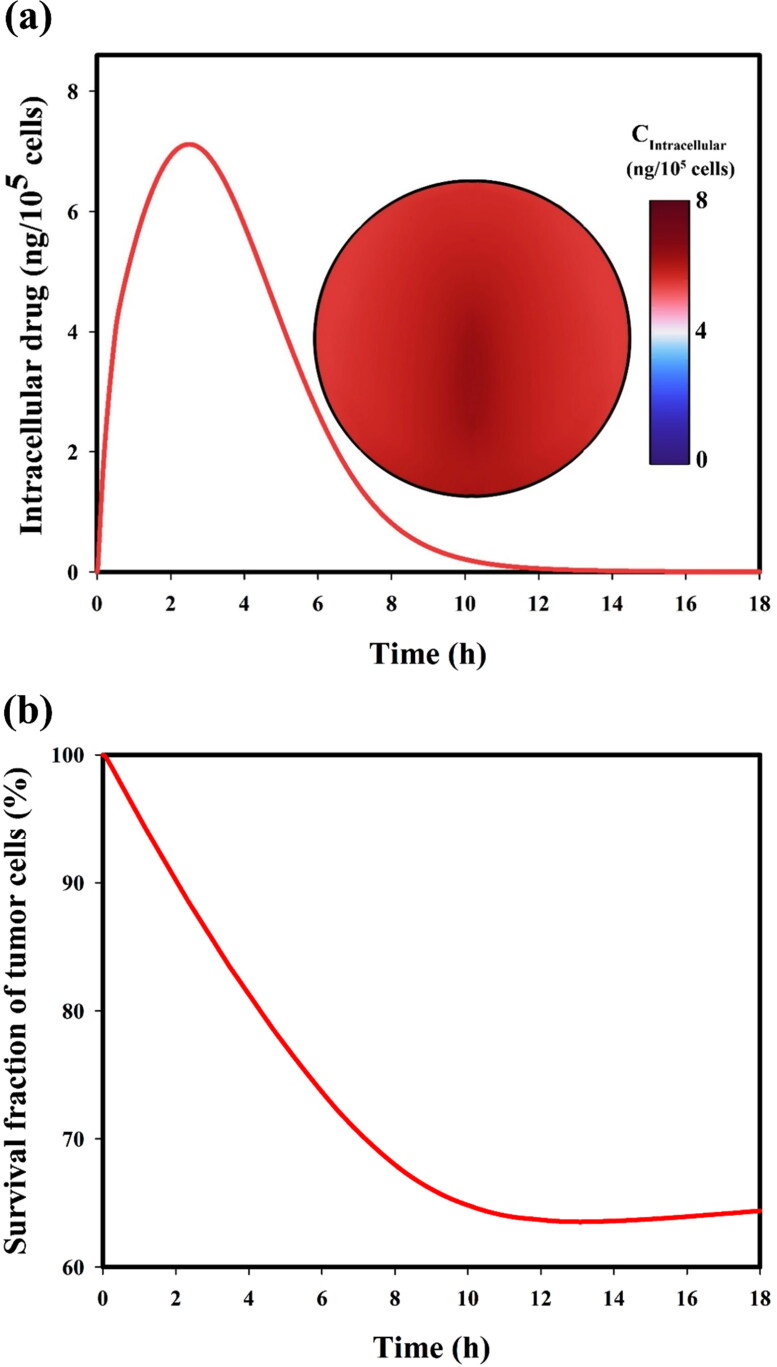
(a) Drug concentration in intracellular space, and (b) survival fraction of tumor cells over time.

Time-dependent variation of the drug’s intracellular concentration and tumor cells’ survival fraction are shown in Figure 9. Concentration in intracellular space increases to its maximum value at about 3 h post-administration of DOX-loaded TSL. Then it gradually decreases to zero as time proceeds (Figure 9(a)). The transmembrane rate (i.e. the rate of drug intake by the cell) is weaker than the diffusion of the drug. Therefore, unused drugs are spread throughout the tumor over time, creating a high concentration of intracellular drug within the tumor after a few hours, while free drug reaches its peak after only half an hour. The maximum of intracellular drug concentration occurs a few hours after the extracellular space drug concentration peak because of the relatively slow transmembrane rate. The efficacy of treatment is assessed with regard to dynamic cell density predicted *via* a pharmacodynamics model (see Table 1, part 5). As shown in Figure 9(b), the survival fraction of cancer cells begins to decrease post-administration immediately, but after 13 h, drug-induced cell killing declines until 15 h when the cell killing rate drops to zero, and proliferation of cells overcomes cell killing.

### 4.6. Effects of drug release rate from nanocarriers and FUS exposure time

Advanced DDSs with precise control over release kinetics are needed for next-generation therapies (Kashkooli et al., 2020; Sarmadi et al., 2022). According to biological, physicochemical, and mathematical principles, various methods can be used for spatiotemporal controlled release and delivery of therapeutic agents, which is necessary for optimizing DDSs. This will lead to increased treatment efficacy, enhanced patient convenience, and improved safety. Encapsulating drugs in TSLs offers great bioavailability and lowers side effects. The intravascular drug release provides a significant drug level in a comparatively small tumor volume. In the current study, drugs are released in the intravascular space, so nanocarriers do not need to aggregate in the tissue’s extracellular matrix. In less than a minute, intravenous administrations expose the entire body to TSLs (Dewhirst & Secomb, 2017). Here, the main question is, ‘what are the effects of different drug release rates on treatment efficacy?’. Therefore, we chose three different release rates of DOX from TSLs in response to heat induced by FUS, namely ultra-fast, fast, and slow release rates (see Table 3 for details).

On the other hand, optimizing the FUS exposure time for intravascular drug release requires in-depth consideration of different factors. Since TSL circulation lasts for roughly 5 h (Gasselhuber et al., 2012; Liu & Xu, 2015), this study also aims to answer another question: ‘what is the optimum exposure time of applying FUS for intravascular release of drug?’. The importance of this parameter is that if the period is too prolonged, the patient’s convenience may be an issue, and if it is too short, the treatment approach may not reach its maximum therapeutic potential. Therefore, an optimized exposure time should be chosen. Various regimens, including continuous exposure of FUS for 10, 30, and 60 min following intravenous injection of TSLs, were investigated.

In this DDS, one of the main challenge is to maintain the needed temperature rise (39.5 °C to 43 °C for ThermoDox) over the whole tumor domain for a time period consistent with the therapeutic agents’ circulating duration (30–60 min when considering a long-circulating TSLs such as ThermoDox) (Lyon et al., 2017; 2021). As typically the temperature profile highly depends on the acoustic power, in this study due to the specific controller design, changing the acoustic power has no effect on the target temperature profile. To achieve an appropriate hyperthermia temperature range during the simulation, a feedback temperature controller is employed to regulate the input power based on the temperature specified in the region of interest (*T_set_* in Table 1) (Gasselhuber et al., 2012; Rezaeian et al., 2019; Zhan et al., 2019; Staruch et al., 2011). This controller’s function is to prevent tumor site temperature from rising above 43 °C in order to protect neighboring healthy tissues from irreversible damage.

**Figure 10.**
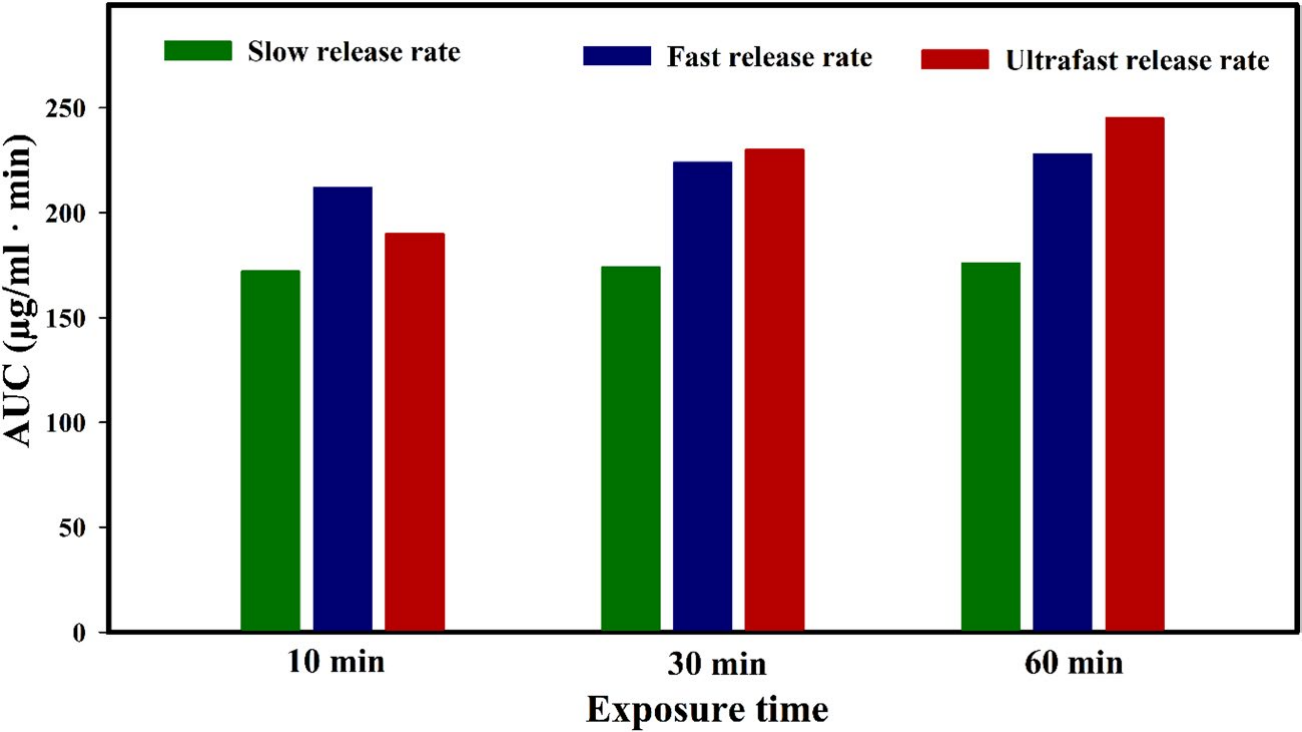
AUC parameter values for the different FUS exposure times using TSL with different drug release rates.

In a preclinical setting, only a few studies have looked at thermal procedures, their combination with temperature-induced drug transport and delivery, and their influence on cancer treatment (Hijnen et al., 2017). Therefore, we have been examined nine scenarios to study the effects of drug release rate and simultaneously FUS exposure time (Figure 10). Based on the literature, for clinical use, hyperthermia for 30 to 60 min following TSL injection appears to be an appropriate balance between achievable amounts of drug, technical challenges with maintaining controlled hyperthermia, and patient comfort (Grüll & Langereis, 2012; Jain et al., 2018). In general, the AUC of the free drug increases when the treatment time is prolonged, maintaining the concentration at a greater level. In 10 min exposure time of FUS, the AUC value is less than 30 and 60 min, because this time is too short for FUS exposure. But for 30 and 60 min FUS exposure, in which the exposure time is long enough for drug release, the higher the release rate, the higher the AUC value. The slow release rate always has the lowest amount of AUC for the different exposure times and scenarios. In the 10 min exposure, the ultrafast release rate provided a lower AUC than the fast release because it is an optimal pattern for intravascular release and has the lowest release rate in the body temperature. However, in 30 and 60 min FUS exposures, the ultrafast release has the highest AUC compared to fast and slow release rates. Although 60 min has a higher AUC compared to 30 min (251 *vs*. 233 [µg/mL·min]), 30 min of FUS exposure is more practical in the clinic in terms of patient comfort and clinical limitations. In addition, the difference in AUC value is not large. It should also be noted that there is no statistically significant variation in AUC between the various cases for slow release rate at the different FUS exposure times. Consequently, a useful therapeutic outcome is provided by 30 min of FUS exposure combined with ultrafast drug release from TSLs (Figure 10).

### 4.7. Sensitivity analysis on drug transport parameters

**Figure 11.**
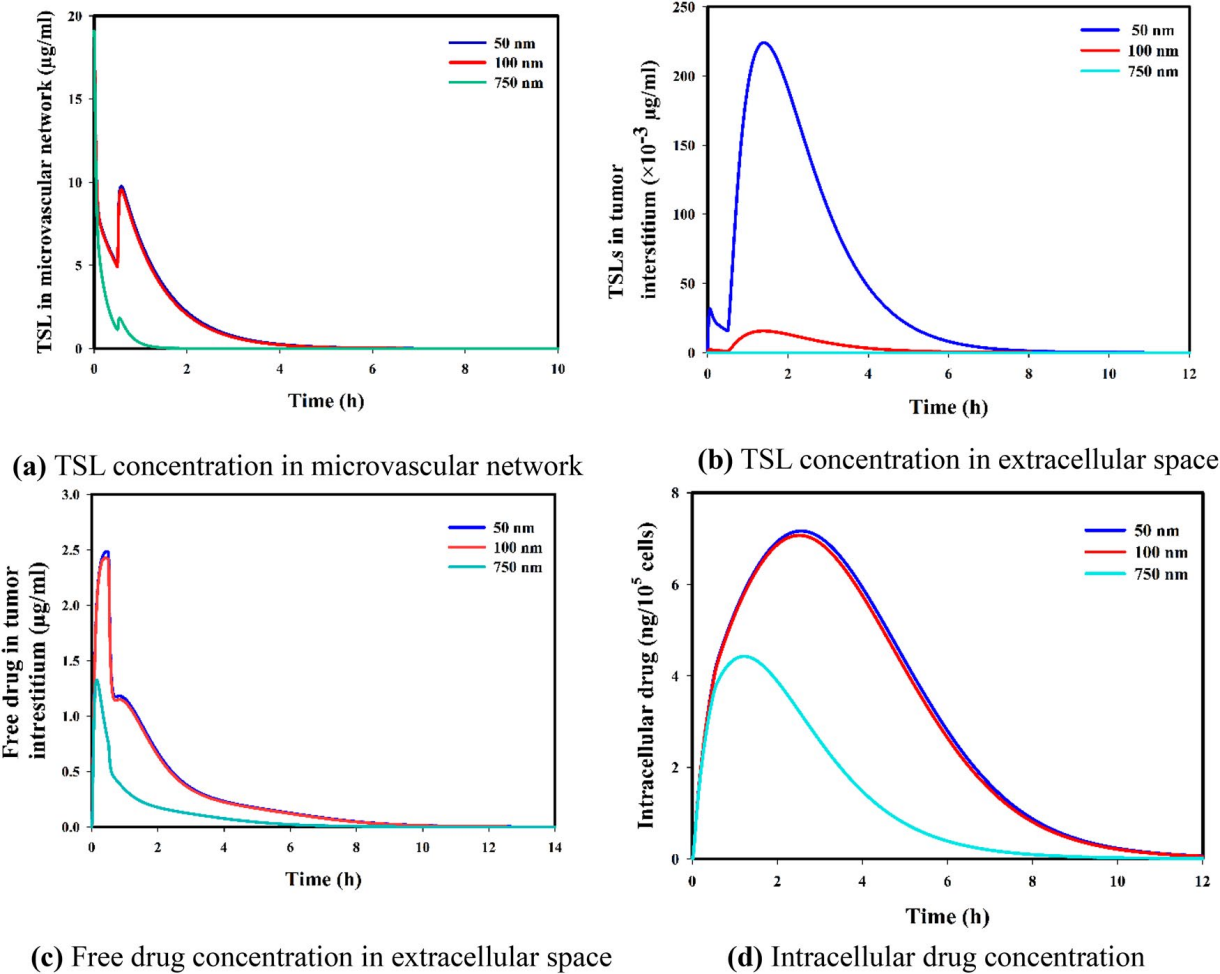
Sensitivity analysis on nanoparticle size (50, 100, 750 nm). (a) TSL concentration in microvascular network, (b) TSL concentration in extracellular space, (c) Free drug concentration in extracellular space, and (d) Intracellular drug concentration.

**Figure 12.**
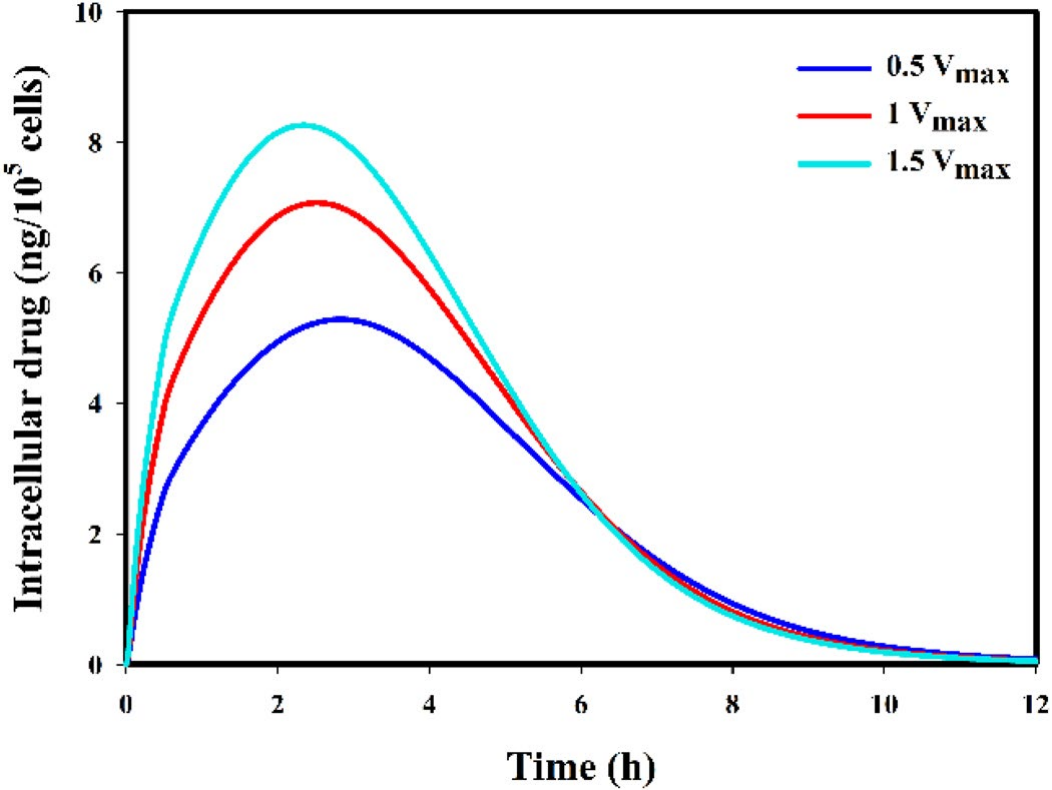
Sensitivity analysis on the transmembrane rate of DOX (a 50% increase or a 50% decrease).

In this section, we have conducted a further sensitivity analysis on the effective parameters to determine which diffusion and transmembrane rate had the highest impact on the concentration profiles. In Figure 11, we have added the results of the sensitivity analysis on nanoparticle size, which is inversely related to the diffusion coefficient in interstitial space (Stylianopoulos et al., 2015; Kashkooli et al., 2022). Small nanoparticles have greater diffusion and accumulate better in the extracellular space; however, the released drugs within the microvascular network mainly impact free drug concentration in extracellular and intracellular spaces almost equally for 50 nm and 100 nm sizes. Here, we have assumed that the loading capacity for the 50 nm and 100 nm nanoparticles is the same, even though published evidence suggests that 100 nm nanoparticles have a higher loading capacity. Larger nanoparticles (for example, 750 nm) are removed quickly due to their higher clearance rate; hence, it causes a poor therapeutic response. On the other hand, the effect of the transmembrane rate of DOX (50% increase or decrease) on intracellular concentration level is shown in Figure 12. Results clearly showed that the higher the value of the transmembrane rate of the drug, the higher the concentration level.

### 4.8. Potentials, limitations and future works

Computational models could offer a more complete description of nano-bio interactions, which is an important challenge for understanding larger-scale processes at the mechanistic level. In *in vivo* models, how drugs are distributed and delivered and their impact on cell death is challenging to explain empirically. Approaches such as mathematical modeling cannot capture all processes in an *in vivo* model, particularly spatial distribution variations in tumor tissue. Using computational models to assess therapeutic agent behavior at various transport phases, combined with limited animal model data, is a promising approach. In summary, while mathematical models are valuable tools, they cannot be parameterized and validated without complementary empirical measurements and thus cannot reach their full potential. *In vivo* models, on the other hand, are difficult to use in the study of nano-bio interactions, and it is difficult to gather quantitative information. As a result, linking computational and *in vivo* models could assist in clinical translation (Kashkooli et al., 2021). In the case of the present study, we have presented a comprehensive multi-scale and multi-physics computational model to investigate different transport phenomena starting from the injection to transport in different environments like the microvasculature and interstitial space and cells, and finally delivery to the cells. Additionally, we have modeled the ultrasound beam propagation physics and its effect on the tissue through the BHTE and evaluated the effect of different exposure parameters. This model can serve as a platform for analyzing the problem of ultrasound-activated nanosized DDS, developing new models, and for the qualitative and quantitative examination of ultrasound-mediated drug delivery to solid tumors.

Mathematical modeling, as a cost-efficient approach, can facilitate the analysis process through complete parametric studies to determine the most effective parameters and optimize their ranges to provide a guideline for the efficient design of both drug-loaded TSLs as treatment plans. However, four major assumptions have been made in the present study, including (*i*) A 2D-axisymmetric simulation model is utilized because of the computational cost of 3D models; (*ii*) Physical complexities of acoustic waves such as cavitation and non-linear propagation are not taken into account; (*iii*) Effects of temperature on tumor characteristics is complex and some of them are not included in the present study (e.g. the dependence of plasma clearance on temperature); (*iv*) Focal area is ideally located in the tumor, regardless of the actual geometry of microvasculature and some other characteristics of TME like necrotic core; (*v*) Main focus of the current study is on the thermal effects of ultrasound and any mechanical impacts (including sonoporation) were not explored. In a domain with uniform distribution of microvessels, a 2D-axisymmetric model can provide reasonable results. However, owing to the complexity of the different physics and the number of partial differential equations, 3D modeling is very time-consuming. Due to our model’s complexity and comprehensiveness, it includes many partial differential equations, tumor parameters, drug properties, and ultrasound exposure parameters. The proposed model has been verified in various parts, but since averaged and representative parameter values have been utilized in the numerical modeling, the results of this study can be mainly used for qualitative analysis instead of quantitative prediction. Multiple studies in the literature also demonstrated that for the focal intensities between 100 and 1000 W/cm^2^, the maximum negative pressure between 1 and 4 MPa, the complications like cavitation and highly non-linear propagation could be ignored with reasonable errors (Hallaj & Cleveland, 1999; Filonenko & Khokhlova, 2001; Sheu et al., 2011).

One of the most important concerns in intravascular drug release is the possibility of drugs penetrating surrounding healthy tissues. However, the drug concentration in the circulatory system in the present study is in a low range, which the published literature (Ten Hagen et al., 2021; Jadidi et al., 2022) suggests does not cause serious side effects (Figure S1). In Figure S1, we have also compared our results for the intravascular drug release paradigm with Souri et al. (Souri et al., 2021) for free chemotherapy, where the maximum of the drug in microvasculature is lower than for free chemotherapy. This reveals a lower potential of drug penetration into healthy tissues when compared to for free chemotherapy. It should also be mentioned that at some point, the concentration of DOX would be higher in tumor tissue, leading to a backflush of Dox to the vessels through diffusion. Indeed, due to the exchange term ($P_{L}\frac{S}{V}(C_{\mathrm{LTP}}-C_{l})\frac{{Pe}_{l}}{e^{{Pe}_{l}}-1}$), backflush to the bloodstream is also possible as the *Pe* number includes diffusion and convection terms. On the other hand, TSL compositions may be unstable at normal body temperatures in the human circulatory system (Tagami et al., 2011). The ideal TSL should be capable of releasing its contents only in response to a few degree temperature raise (Zou et al., 1993). Drug release at normal body temperature can result in the accumulation of the free drug in the bloodstream damaging healthy cells and increasing systemic side effects, including cardiotoxicity (Legha et al., 1982). Hence, future studies on developing TSLs should aim to minimize leakage of drug at normal body temperature, improve the release rate of drug from TSLs at predetermined temperature, and optimize hyperthermia treatment planning (Liu & Xu, 2015). For future works of our group, low-intensity pulsed ultrasound (LIPUS) and FUS are two therapeutic ultrasound exposure approaches which will be used to activate drugs from two common nanocarriers (ThermoDox and gold nanoparticles), both theoretically and experimentally. LIPUS transducers normally produce unfocused ultrasound beams with a low acoustic intensity range. In comparison, FUS’s focal intensity varies from a few W/cm^2^ to thousands W/cm^2^. It should be noted that ThermoDox’s primary mechanism governing drug release is temperature driven, while for gold nanocarriers, drug release is governed by both thermal and mechanical mechanisms. Additionally, ultrasound-activated DDSs using different administration approaches (e.g. intratumoral and intraperitoneal) of therapeutic agents will also be investigated.

## 5. Conclusions

Targeted DDSs using TSLs and an external field like ultrasonic energy for activating drug release is a promising therapeutic approach. However, several factors, including the formulation of the DDSs, the properties of the drugs, and the characteristics of the cancer cells and TME, influence the successful designs of DDSs. A multi-scale computational model of FUS-triggered nano-sized DDS to solid tumors is proposed in this study to evaluate treatment efficacy. The main aim of the presented computational modeling is to study and understand the significant interactions between the TME and FUS-triggered nano-sized DDS. The spatiotemporal distribution of drug-loaded TSLs, the temperature response of FUS heating, and cellular uptake of drug for a combination of FUS and DOX-loaded TSL delivery systems are studied through simulations. First, the acoustic and thermal fields are simulated. Using the temperature gradient in the tumor, drugs are released in different areas at a specific rate. The drug transport process in various tumor compartments is taken into account using fully-coupled equations of fluid flow in the interstitium and mass transport in tissue and cellular spaces. Eventually, distributions of acoustic and thermal fields, IFV, IFP, various concentrations, AUC, and the fraction of survival cells post-treatment are determined according to physiological data and biological factors. Results demonstrate that, in an intravenous administration of TSLs for intravascular release of drug, treatment should be prolonged until achieving the maximum free drug concentration. This will maximize tumor cell killing. The maximum concentration in the current study was reached at about 25 min. Applying 30 min FUS is the best option among different cases in terms of both therapeutic efficacy and patient comfort because it is more realistic and feasible for clinical trials. Additionally, the ultrafast release of drug DOX from TSLs has shown better compatibility with intravascular drug release, leading to higher therapeutic efficacy. Results also show the ability of the current approach to model a complex system, demonstrating this DDS’s capability for achieving a localized, targeted therapy along with improved delivery of drug in TME while maintaining a relatively small drug concentration level in healthy tissue. The study demonstrates how mathematical modeling and computational tools can be created synergistically to simulate the role of FUS in drug release from TSLs and drug transport in tumor tissue. The proposed framework for mathematical modeling can serve as a platform for future studies on FUS heat-induced nano-sized DDSs.

## Supplementary Material

Supplemental Material

## Data Availability

The data supporting this work are accessible upon reasonable request from the corresponding author.

## List of Abbreviations

AUC: area under the curve
BC: boundary condition
BHTE: bio-heat transfer equation
CDR: convection-diffusion-reaction
DDSs: drug delivery systems
DOX: doxorubicin
EPR: enhanced permeability and retention
FUS: focused ultrasound
HIFU: high intensity focused ultrasound
IFP: interstitial fluid pressure
IFV: interstitial fluid velocity
LATS: linear acoustic and temperature simulator
LIPUS: low-intensity pulsed ultrasound
NPs: nanoparticles
PI: proportional–integral
PML: perfectly matched layer
TME: tumor microenvironment
TSLs: temperature-sensitive liposomes
