# Supplementary material for: A spatiotemporal computational model of focused ultrasound heat-induced nano-sized drug delivery system in solid tumors
Source: Drug Deliv. 2023 Jun 14;30(1):2219871. doi: 10.1080/10717544.2023.2219871 (PMC10269399; doi:10.1080/10717544.2023.2219871)
Supplement: Supplemental Material [file IDRD_A_2219871_SM9654.docx]

**Supporting file**

**A Spatiotemporal Computational Model of Focused Ultrasound Heat-Induced Nano-Sized Drug Delivery System into Solid Tumors**

| **Table S1.** Physiological parameters. | | | | | | | |
| --- | --- | --- | --- | --- | --- | --- | --- |
| Parameter | Definition | Unit | Tumor tissue | Normal tissue | Blood | Whole  body | Ref. |
| $S/V$ | Surface area of blood vessels per unit tissue volume | $m^{-1}$ | 20000 | 7000 | - | - | [1, 2] |
| $K_{v}$ | Hydraulic conductivity of the wall of microvessels | $m/Pa\cdot s$ | $2.1\times{10}^{-11}$ | $2.7\times{10}^{-12}$ | - | - | [1, 2] |
| $\rho$ | Density (at 37 $℃$) | $kg/m^{3}$ | 1000 | 1000 | 1060 | - | [3-5] |
| $\mu$ | Dynamic viscosity of interstitial fluid | $kg/m\cdot s$ | $7.8\times{10}^{-4}$ | $7.8\times{10}^{-4}$ | - | - | [6] |
| $\frac{1}{\kappa}$ | Permeability of the interstitial space | $m^{-2}$ | $4.56\times{10}^{16}$ | $2.21\times{10}^{17}$ | - | - | [1, 6] |
| $p_{v}$ | Vascular fluid pressure | $Pa$ | 2080 | 2080 | - | - | [1, 7, 8] |
| $\pi_{v}$ | Osmotic pressure of the plasma | $Pa$ | 2666 | 2666 | - | - | [1, 7, 8] |
| $\pi_{i}$ | Osmotic pressure of interstitial fluid | $Pa$ | 2000 | 1333 | - | - | [1, 7, 8] |
| $\sigma_{T}$ | Average osmotic reflection coefficient | 1 | 0.82 | 0.91 | - | - | [1, 7, 8] |
| $K_{ly}S_{ly}/V$ | Hydraulic conductivity of the lymphatic wall times surface area of lymphatic vessels per unit volume of tumor tissue | $\frac{1}{(pa\cdot s)}$ | 0 | $4.17\times{10}^{-7}$ | - | - | [6] |
| $p_{ly}$ | Intra-lymphatic pressure | $Pa$ | 0 | 0 | - | - | [6] |
| $D_{c}$ | Cell density | ${10}^{5}cell/m^{3}$ | ${10}^{10}$ | ${10}^{10}$ | - | - | [9, 10] |
| $c_{0}$ | Ultrasound speed (at 37 $℃$) | $m/s$ | 1600 | 1600 | 1540 | - | [4, 5] |
| $c$ | Specific heat (at 37 $℃$) | $J/(kg\cdot K)$ | 3800 | 3600 | 3770 | - | [4] |
| $k$ | Thermal conductivity (at 37 $℃$_0_) | $W/(m\cdot K)$ | 0.552 | 0.512 | 0.53 | - | [4] |
| $w_{b0}$ | Blood perfusion rate (at 37 $℃$) | $s^{-1}$ | 0.002 | 0.018 | - | - | [11] |
| $V_{BB}$ | Total blood volume in body | l | - | - | - | 5.53 | [12] |
| $H_{ct}$ | Hematocrit | 1 | 0.19 | 0.45^*^ | - | 0.45 | [13, 14] |
| $V_{TV}$ | Volume fraction of tissue vascular space | 1 | 0.092 | 0.0322^*^ | - | - | [15] |
| $V_{BT}$ | Volume of body tissue | l | - | - | - | 64.47 | [12] |
| $Q_{m}$ | The heat generated by metabolism | $\frac{W}{m^{-3}}$ | 0 | 0 | - | - | [16, 17] |
| * The related values are assumed in this study. | | | | | | | |

| **Table S2.** Parameters for DOX | | | | | |
| --- | --- | --- | --- | --- | --- |
| Parameter | Definition | Unit | Free DOX | Bound DOX | Ref. |
| $P_{tumor}$ | Permeability of vasculature wall in tumor tissue | $m/s$ | $3.6\times{10}^{-6}$ | $7.8\times{10}^{-9}$ | [6, 18] |
| $P_{\mathrm{normal}}$ | Permeability of vasculature wall in normal tissue | $m/s$ | $3.75\times{10}^{-7}$ | $2.5\times{10}^{-9}$ | [6, 18] |
| $D_{tumor}$ | Diffusion coefficient in interstitial fluid of tumor | $m^{2}/s$ | $3.4\times{10}^{-10}$ | $8.89\times{10}^{-12}$ | [6, 19-21] |
| $D_{\mathrm{normal}}$ | Diffusion coefficient in interstitial fluid of normal | $m^{2}/s$ | $1.58\times{10}^{-10}$ | $4.17\times{10}^{-12}$ | [6, 19-21] |
| $\sigma_{d}$ | Osmotic reflection coefficient | 1 | 0.15 | 0.82 | [22] |
| $k_{a}$ | Doxorubicin-protein binding rate | $s^{-1}$ | 0.833 | - | [9] |
| $k_{d}$ | Doxorubicin-protein dissociation rate | $s^{-1}$ | - | 0.278 | [9] |
| $\varphi$ | Tumor fraction extracellular space | 1 | 0.4 | - | [9] |
| $V_{max}$ | Rate of trans-membrane transport | $kg/{10}^{5}cell\cdot s$ | $4.67\times{10}^{-15}$ | - | [9, 23] |
| $k_{e}$ | Michaelis constant for transmembrane transport | $kg/m^{3}$ | $2.19\times{10}^{-4}$ | - | [9, 23] |
| $k_{i}$ | Michaelis constant for transmembrane transport | $kg/{10}^{5}cells$ | $1.37\times{10}^{-12}$ | - | [9, 23] |
| $f_{max}$ | Cell-kill rate constant | $s^{-1}$ | $1.67\times{10}^{-5}$ | - | [24] |
| ${EC}_{50}$ | Drug concentration producing 50% of $f_{max}$ | $kg/{10}^{5}cells$ | $5\times{10}^{-13}$ | - | [24] |
| $k_{c}$ | Cell proliferation rate | $s^{-1}$ | $3\times{10}^{-6}$ | - | [25] |
| $k_{g}$ | Cell physiologic degradation rate | $s^{-1}$ | $3\times{10}^{-16}$ | - | [25] |
| ${CL}_{Tumor}$ | Plasma clearance in tissue | $s^{-1}$ | $2.43\times{10}^{-3}$ | 0 | [26, 27] |
| ${CL}_{Sys}$ | Plasma clearance in Systemic | $s^{-1}$ | $1.1 \times{10}^{-3}$ | 0 | [28] |
| $k_{P}$ | Transfer constant free drug from systemic plasma to tissue | $s^{-1}$ | $1.6 \times{10}^{-3}$ | $9.6 \times{10}^{-5}$^*^ | [28] |
| $k_{t}$ | Transfer constant free drug from tissue to systemic plasma | $s^{-1}$ | $4.8 \times{10}^{-5}$ | $2.8 \times{10}^{-7}$^*^ | [28] |
| * The related values are assumed in this study. | | | | | |

| **Table S3.** Parameters for TSLs | | | | | | |
| --- | --- | --- | --- | --- | --- | --- |
| Parameter | Definition | Unit | Tumor tissue | Normal tissue | Systemic | Ref. |
| $P_{L}$ | Liposome permeability of vasculature wall | $m/s$ | $5.44\times{10}^{-12}$ | $0$^*^ | - | [29] |
| $D_{L}$ | Hydraulic conductivity of the microvascular wall | $m^{2}/s$ | $2.2\times{10}^{-12}$ | $1.41\times{10}^{-12}$ | - | [29] |
| $\sigma_{L}$ | Reflection coefficient for liposome | 1 | 95 | 1 | - | [17] |
| ${CL}_{Lipt}$ | TSL clearance in tissue plasma | $s^{-1}$ | $2.228\times{10}^{-4}$ | $2.228\times{10}^{-4}$ | - | [30] |
| ${CL}_{Lips}$ | TSL clearance in systemic plasma | $s^{-1}$ | - | - | $9.417\times{10}^{-6}$ | [12] |
| * The related values are assumed in this study. | | | | | | |

| **Table S4.** Acoustic parameters | | | | |
| --- | --- | --- | --- | --- |
| Parameter | Definition | Unit | Value | Ref. |
| *f* | Ultrasound frequency | MHz | 2 | - |
| $\alpha$ | Local acoustic  absorption coefficient | Np/m | $\alpha_{0} \left( \frac{f}{f_{0}} \right)^{1}=17.96$; For tumor and normal tissue $\alpha_{0} \left( \frac{f}{f_{0}} \right)^{2}=0.1013$; For water & $f_{0}=1$ | [5, 31] |
| *c* | Speed of sound (for water) | m/s | 1520.6 | [5] |
| $\rho$ | Density (for water) | $kg/m^{3}$ | 994.23 | [5] |

| 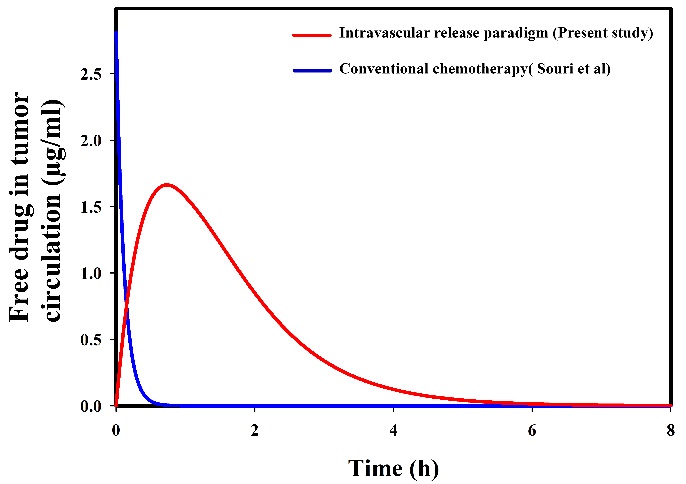 | 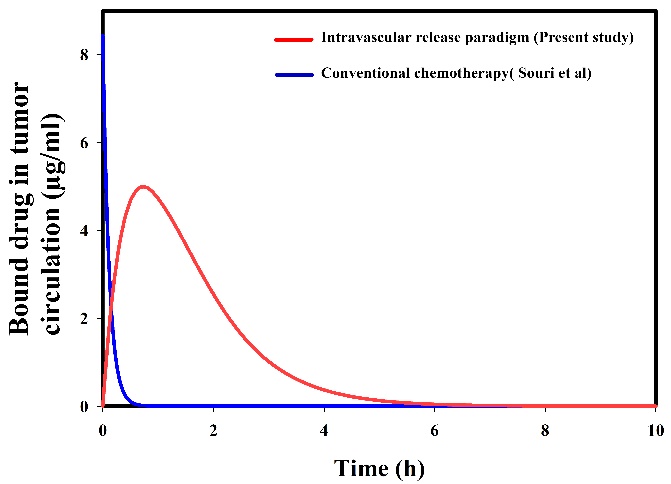 |
| --- | --- |
| (a) Free drug | (b) Protein-bound drug |
| **Fig. S1.** Free and protein-bound drug concentrations in the circulatory system | |

**Reference**

1. Baxter, L.T. and R.K. Jain, *Transport of fluid and macromolecules in tumors. I. Role of interstitial pressure and convection.* Microvascular research, 1989. **37**(1): p. 77-104.

2. Soltani, M. and P. Chen, *Numerical modeling of fluid flow in solid tumors.* PloS one, 2011. **6**(6): p. e20344.

3. Kashkooli, F.M., et al., *Effect of vascular normalization on drug delivery to different stages of tumor progression: In-silico analysis.* Journal of Drug Delivery Science and Technology, 2020. **60**: p. 101989.

4. Sheu, T.W., et al., *On an acoustics–thermal–fluid coupling model for the prediction of temperature elevation in liver tumor.* International Journal of Heat and Mass Transfer, 2011. **54**(17-18): p. 4117-4126.

5. Duck, F.A., *Physical properties of tissues: a comprehensive reference book*. 2013: Academic press.

6. Goh, Y.-M.F., H.L. Kong, and C.-H. Wang, *Simulation of the delivery of doxorubicin to hepatoma.* Pharmaceutical Research, 2001. **18**(6): p. 761-770.

7. Baxter, L.T. and R.K. Jain, *Transport of fluid and macromolecules in tumors. II. Role of heterogeneous perfusion and lymphatics.* Microvascular research, 1990. **40**(2): p. 246-263.

8. Baxter, L.T. and R.K. Jain, *Transport of fluid and macromolecules in tumors. IV. A microscopic model of the perivascular distribution.* Microvascular research, 1991. **41**(2): p. 252-272.

9. Eikenberry, S., *A tumor cord model for doxorubicin delivery and dose optimization in solid tumors.* Theoretical Biology and Medical Modelling, 2009. **6**(1): p. 16.

10. Zhan, W., *Mathematical modelling of drug delivery to solid tumour.* 2014.

11. Vaupel, P., F. Kallinowski, and P. Okunieff, *Blood flow, oxygen and nutrient supply, and metabolic microenvironment of human tumors: a review.* Cancer research, 1989. **49**(23): p. 6449-6465.

12. Gasselhuber, A., et al., *Targeted drug delivery by high intensity focused ultrasound mediated hyperthermia combined with temperature-sensitive liposomes: computational modelling and preliminary in vivo validation.* International Journal of Hyperthermia, 2012. **28**(4): p. 337-348.

13. Tofts, P.S., et al., *Estimating kinetic parameters from dynamic contrast‐enhanced T1‐weighted MRI of a diffusable tracer: standardized quantities and symbols.* Journal of Magnetic Resonance Imaging: An Official Journal of the International Society for Magnetic Resonance in Medicine, 1999. **10**(3): p. 223-232.

14. Brizel, D.M., et al., *A comparison of tumor and normal tissue microvascular hematocrits and red cell fluxes in a rat window chamber model.* International Journal of Radiation Oncology* Biology* Physics, 1993. **25**(2): p. 269-276.

15. Yuan, F., et al., *Microvascular permeability of albumin, vascular surface area, and vascular volume measured in human adenocarcinoma LS174T using dorsal chamber in SCID mice.* Microvascular research, 1993. **45**(3): p. 269-289.

16. Rezaeian, M., A. Sedaghatkish, and M. Soltani, *Numerical modeling of high-intensity focused ultrasound-mediated intraperitoneal delivery of thermosensitive liposomal doxorubicin for cancer chemotherapy.* Drug delivery, 2019. **26**(1): p. 898-917.

17. Zhan, W., W. Gedroyc, and X.Y. Xu, *Towards a multiphysics modelling framework for thermosensitive liposomal drug delivery to solid tumour combined with focused ultrasound hyperthermia.* Biophysics Reports, 2019. **5**(1): p. 43-59.

18. Wu, N.Z., et al., *Measurement of material extravasation in microvascular networks using fluorescence video-microscopy.* Microvascular research, 1993. **46**(2): p. 231-253.

19. Jain, R.K., *Transport of molecules in the tumor interstitium: a review.* Cancer research, 1987. **47**(12): p. 3039-3051.

20. Swabb, E.A., J. Wei, and P.M. Gullino, *Diffusion and convection in normal and neoplastic tissues.* Cancer research, 1974. **34**(10): p. 2814-2822.

21. Nugent, L.J. and R.K. Jain, *Extravascular diffusion in normal and neoplastic tissues.* Cancer research, 1984. **44**(1): p. 238-244.

22. Wolf, M.B., P.D. Watson, and D. Scott 2nd, *Integral-mass balance method for determination of solvent drag reflection coefficient.* American Journal of Physiology-Heart and Circulatory Physiology, 1987. **253**(1): p. H194-H204.

23. Kerr, D.J., et al., *Comparative intracellular uptake of adriamycin and 4'-deoxydoxorubicin by nonsmall cell lung tumor cells in culture and its relationship to cell survival.* Biochemical pharmacology, 1986. **35**(16): p. 2817-2823.

24. Eliaz, R.E., et al., *Determination and modeling of kinetics of cancer cell killing by doxorubicin and doxorubicin encapsulated in targeted liposomes.* Cancer research, 2004. **64**(2): p. 711-718.

25. Liu, C., J. Krishnan, and X.Y. Xu, *Investigating the effects of ABC transporter-based acquired drug resistance mechanisms at the cellular and tissue scale.* Integrative Biology, 2013. **5**(3): p. 555-568.

26. Benet, L.Z. and P. Zia-Amirhosseini, *Basic principles of pharmacokinetics.* Toxicologic pathology, 1995. **23**(2): p. 115-123.

27. Rodvold, K.A., D.A. Rushing, and D.A. Tewksbury, *Doxorubicin clearance in the obese.* Journal of Clinical Oncology, 1988. **6**(8): p. 1321-1327.

28. Gasselhuber, A., et al., *Mathematical spatio-temporal model of drug delivery from low temperature sensitive liposomes during radiofrequency tumour ablation.* International Journal of Hyperthermia, 2010. **26**(5): p. 499-513.

29. Stylianopoulos, T., et al., *Towards optimal design of cancer nanomedicines: Multi-stage nanoparticles for the treatment of solid tumors.* Annals of biomedical engineering, 2015. **43**(9): p. 2291-2300.

30. Gabizon, A., et al., *Prolonged circulation time and enhanced accumulation in malignant exudates of doxorubicin encapsulated in polyethylene-glycol coated liposomes.* Cancer research, 1994. **54**(4): p. 987-992.

31. Hornsby, T., E. Shaswary, and J. Tavakkoli, *Development of an ultrasonic nonlinear frequency compounding method with applications in tissue thermometry.* The Journal of the Acoustical Society of America, 2021. **150**(4): p. 3192-3203.
